# NGL-3 in the regulation of brain development, Akt/GSK3b signaling, long-term depression, and locomotive and cognitive behaviors

**DOI:** 10.1371/journal.pbio.2005326

**Published:** 2019-06-05

**Authors:** Hyejin Lee, Wangyong Shin, Kyungdeok Kim, Suho Lee, Eun-Jae Lee, Jihye Kim, Hanseul Kweon, Eunee Lee, Haram Park, Muwon Kang, Esther Yang, Hyun Kim, Eunjoon Kim

**Affiliations:** 1 Center for Synaptic Brain Dysfunctions, Institute for Basic Science (IBS), Daejeon, Korea; 2 Department of Biological Sciences, Korea Advanced Institute for Science and Technology (KAIST), Daejeon, Korea; 3 Department of Neurology, Asan Medical Center University of Ulsan, College of Medicine, Seoul, South Korea; 4 Department of Anatomy, College of Medicine, Korea University, Seoul, Korea; Thomas Jefferson University, United States of America

## Abstract

Netrin-G ligand-3 (NGL-3) is a postsynaptic adhesion molecule known to directly interact with the excitatory postsynaptic scaffolding protein postsynaptic density-95 (PSD-95) and *trans*-synaptically with leukocyte common antigen-related (LAR) family receptor tyrosine phosphatases to regulate presynaptic differentiation. Although NGL-3 has been implicated in the regulation of excitatory synapse development by in vitro studies, whether it regulates synapse development or function, or any other features of brain development and function, is not known. Here, we report that mice lacking NGL-3 (*Ngl3*^*−/−*^ mice) show markedly suppressed normal brain development and postnatal survival and growth. A change of the genetic background of mice from pure to hybrid minimized these developmental effects but modestly suppressed N-methyl-D-aspartate (NMDA) receptor (NMDAR)-mediated synaptic transmission in the hippocampus without affecting synapse development, α-amino-3-hydroxy-5-methyl-4-isoxazolepropionic acid (AMPA) receptor (AMPAR)-mediated basal transmission, and presynaptic release. Intriguingly, long-term depression (LTD) was near-completely abolished in *Ngl3*^*−/−*^ mice, and the Akt/glycogen synthase kinase 3β (GSK3β) signaling pathway, known to suppress LTD, was abnormally enhanced. In addition, pharmacological inhibition of Akt, but not activation of NMDARs, normalized the suppressed LTD in *Ngl3*^*−/−*^ mice, suggesting that Akt hyperactivity suppresses LTD. *Ngl3*^*−/−*^ mice displayed several behavioral abnormalities, including hyperactivity, anxiolytic-like behavior, impaired spatial memory, and enhanced seizure susceptibility. Among them, the hyperactivity was rapidly improved by pharmacological NMDAR activation. These results suggest that NGL-3 regulates brain development, Akt/GSK3β signaling, LTD, and locomotive and cognitive behaviors.

## Introduction

Synaptic adhesion molecules regulate diverse aspects of synapse development, function, and plasticity. A large number of synaptic adhesion molecules have been identified, typified by members of the neuroligin and neurexin families [1–16]. These molecules are thought to regulate neuronal synapses through diverse mechanisms, including *trans*-synaptic adhesions and cytoplasmic interactions with scaffolding and signaling proteins, although in vivo evidence in support of these mechanisms is generally limited.

Netrin-G ligands (NGLs; also known as LRRC4s) are a family of postsynaptic adhesion molecules with three known members: NGL-1/LRRC4C, NGL-2/LRRC4, and Netrin-G ligand-3 (NGL-3)/LRRC4B [17–19]. NGL-3 interacts with presynaptic leukocyte common antigen-related (LAR) family receptor tyrosine phosphatases (LAR-RPTPs; LAR, protein tyrosine phosphatase σ [PTPσ], and protein tyrosine phosphatase δ [PTPδ]) [20, 21], which have been implicated in diverse psychiatric disorders and are known to regulate both early neurodevelopmental processes and postnatal synapse development [1, 4]. In addition to NGL-3, LAR-RPTPs have recently been shown to interact with several other postsynaptic adhesion molecules, including TrkC, interleukin 1 receptor accessory protein like 1 (IL1RAPL1), interleukin 1 receptor accessory protein (IL1RAcP), Slit- and Trk-like proteins (Slitrk1–5), synaptic adhesion-like molecule 3 (SALM3), and SALM5 [19, 21–30]. Although these *trans*-synaptic adhesion complexes constitute a rapidly expanding group of synaptic organizers, little is known about their differential functions. In particular, it is unclear which *trans*-synaptic complexes are more important for early neurodevelopmental processes versus postnatal synapse development.

Intracellularly, NGL-3 directly interacts with postsynaptic density-95 (PSD-95), an abundant excitatory postsynaptic scaffolding protein, through its C-terminal PDZ-binding motif [31], and induces postsynaptic clustering of PSD-95, N-methyl-D-aspartate (NMDA) receptors (NMDARs), and α-amino-3-hydroxy-5-methyl-4-isoxazolepropionic acid (AMPA) receptors (AMPARs) [22], strongly implicating NGL-3 in the regulation of excitatory synapse development and function. For instance, NGL-3 overexpression and knockdown in cultured neurons result in bidirectional changes in the number of excitatory synapses and spontaneous excitatory synaptic transmission [22]. However, whether NGL-3 regulates synapse development in vivo remains unclear. In addition, it is not known whether NGL-3 regulates other synaptic and higher brain functions, such as synaptic transmission, synaptic plasticity, brain excitability, and specific behaviors.

In the present study, we found that *Ngl3/Lrrc4b* knockout (KO) in a pure C57BL/6J genetic background (termed *Ngl3*^*−/−*(B6)^ mice) decreases birth rate, postnatal growth and survival, and brain development, whereas *Ngl3* KO in a hybrid genetic background (*Ngl3*^*−/−*(Hyb)^ mice) does not induce these neurodevelopmental features. Instead, these latter mice display modestly reduced NMDAR- but not AMPAR-dependent synaptic transmission. Intriguingly, long-term depression (LTD) was near-completely abolished in the hippocampus of both *Ngl3*^*−/−*^ mice, and the Akt/glycogen synthase kinase 3β (GSK3β) signaling pathway, known to suppress LTD, was abnormally enhanced. Importantly, Akt inhibition normalized the suppressed LTD in the *Ngl3*^*−/−*^ hippocampus, suggesting that enhanced Akt/GSK3β phosphorylation suppresses LTD and that NGL-3 regulates Akt/GSK3β signaling. Behaviorally, these *Ngl3*^*−/−*(Hyb)^ mice show hyperactivity that is responsive to NMDAR activation, as well as anxiolytic-like behavior and impaired spatial learning and memory. These results implicate NGL-3 in the regulation of brain development and excitability, Akt/GSK3β signaling, LTD, and behaviors in vivo.

## Results

### *Ngl3*^−/−(B6)^ mice show decreased birth rate and suppressed postnatal growth, survival, and brain development

To investigate in vivo functions of NGL-3, we first generated *Ngl3*^*−/−*^ mice in a C57BL/6J background (*Ngl3*^*−/−*(B6)^) by replacing exon 2 of the *Ngl3* gene (which together with exon 3 encodes the entire NGL-3 protein) with a β-galactosidase + neomycin resistance (β-geo) cassette. *Ngl3* KO was confirmed by PCR-based genotyping and immunoblot analysis (**S1 Fig**).

**Fig 1:**
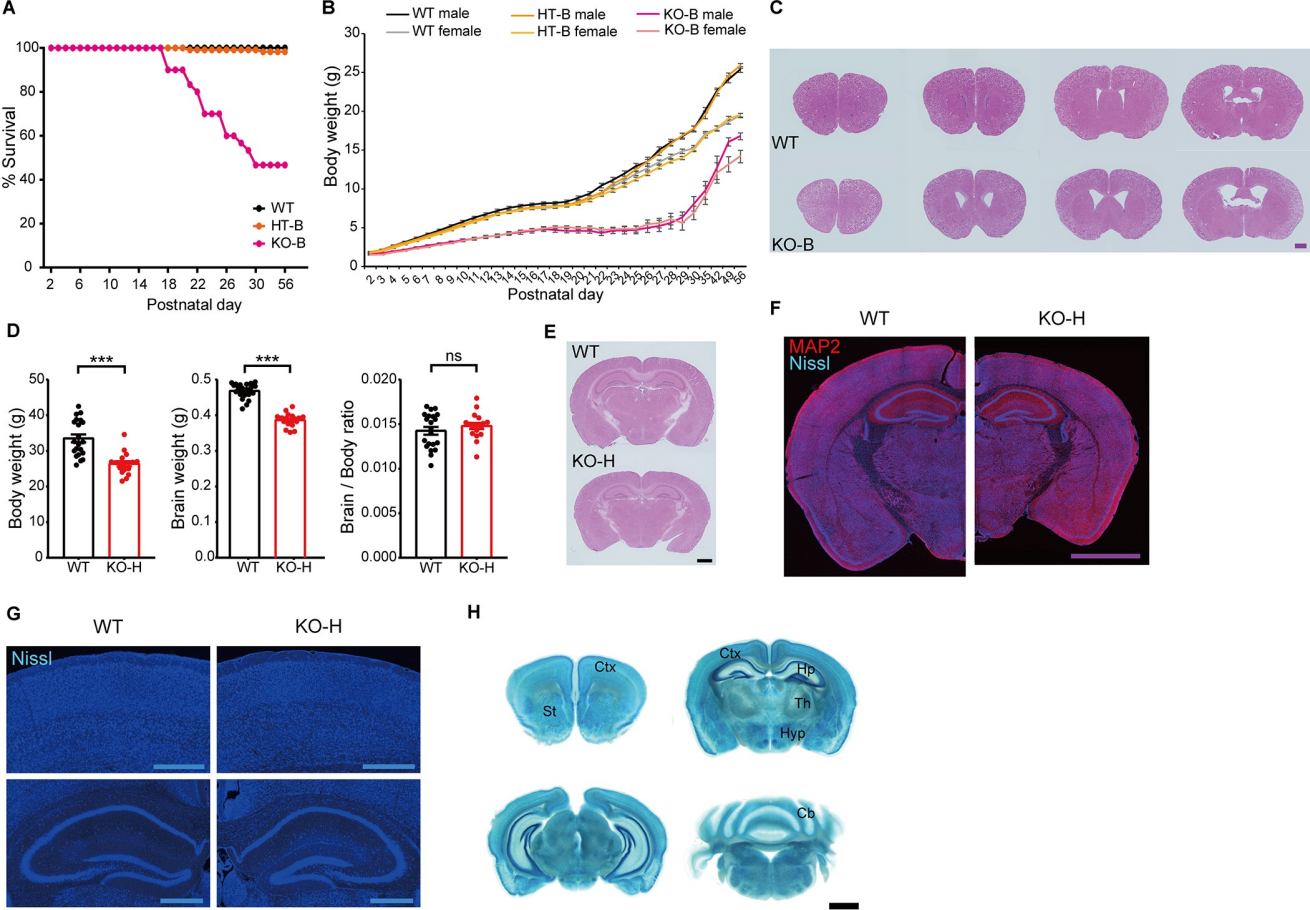
*Ngl3* deletion in mice decreases birth rate and suppresses postnatal growth, survival, and brain development. (A) Reduced survival of *Ngl3*^*−/−*(B6)^ (KO) mice during the first 8 postnatal weeks. *n* = 61 mice for WT, 118 for HT, and 30 for KO. (B) Substantially reduced body weights in *Ngl3*^*−/−*(B6)^ mice compared with WT mice. Note that body weights of *Ngl3*^*+/−*(B6)^ mice are moderately reduced. *n* = 61 mice for WT, 118 for HT, and 30 for KO. (C) Altered gross morphology of the *Ngl3*^*−/−*(B6)^ brain (10 weeks), revealed by HE staining. Note that the sizes of ventricles are increased in *Ngl3*^*−/−*(B6)^ slices. Scale bar, 1 mm. (D) Moderate reductions in both body and brain weights, with unaltered brain/body weight ratios, in *Ngl3*^*−/−*(Hyb)^ mice (3–5 months). KO-H, *Ngl3*^*−/−* (Hyb)^. *n* = 21 mice for WT and 18 for KO, ***P < 0.001, ns, not significant, Student *t* test. (E) Moderately reduced overall size of the *Ngl3*^*−/−*(Hyb)^ brain (10 weeks), as shown by HE staining. Scale bar, 1 mm. (F and G) Reduced brain size in *Ngl3*^*−/−*(Hyb)^ mice (10 weeks), with an apparent decrease in the size of the thalamus, as shown by Nissl (cell body marker) + MAP2 (dendritic marker) staining. Nissl-only images are also shown for clarity. Scale bar, 2 mm for MAP2 and Nissl, and 0.5 mm for Nissl only. (H) NGL-3 expression patterns, as revealed by X-gal staining of *Ngl3*^*−/−*(Hyb)^ brain slices (8–10 weeks). Scale bar, 1 mm. Primary data can be found in S3_Data. Cb, cerebellum; Ctx, cortex; HE, hematoxylin–eosin; Hp, hippocampus; HT, heterozygous; HT-B, heterozygous, C57BL/6; Hyp, hypothalamus; KO, knockout; KO-B, knockout, C57BL/6; KO-H, knockout, hybrid; MAP2, microtubule associated protein 2; NGL-3, Netrin-G ligand-3; ns, not significant; St, striatum; Th, thalamus; WT, wild-type.

*Ngl3*^*−/−*(B6)^ mice showed a reduced Mendelian ratio of approximately 12% compared with the expected approximately 25%, as shown by PCR genotyping performed at approximately postnatal day 7 (P7), suggesting that *Ngl3* KO causes approximately 50% embryonic lethality. In addition, *Ngl3*^*−/−*(B6)^ mice showed reduced postnatal survival, exhibiting a decline in survival rate beginning at about P18 and reaching approximately 50% of wild-type (WT) mice at about P30 (**Fig 1A**). Surviving *Ngl3*^*−/−*(B6)^ mice also showed reduced postnatal growth, as evidenced by decreased whole-body weights (**Fig 1B**) compared with WT and heterozygous *Ngl3*^*+/−*(B6)^ mice. In addition, the timing of weaning had to be shifted about one week (from approximately 3 to 4 weeks) and, after weaning, *Ngl3*^*−/−*(B6)^ mice had difficulty eating food pellets from the overhead grid and usually required the food to be placed on the cage floor.

Hematoxylin–eosin (HE) staining of *Ngl3*^*−/−*(B6)^ brain slices at 10 weeks revealed substantial alterations in the gross morphology of the brain, as exemplified by the enlarged size of lateral and third ventricles (**Fig 1C**). Given these anatomical alterations, we opted not to measure additional details of brain morphology, such as total brain or individual brain-region sizes. These results collectively indicate that *Ngl3* KO in a pure C57BL/6J genetic background decreases birth rate and suppresses postnatal growth, survival, and brain development.

### *Ngl3*^−/−(Hyb)^ mice show normal birth and postnatal growth and survival, and a largely normal brain morphology

To overcome the practical difficulties of producing sufficient numbers of *Ngl3*^*−/−*^ mice for additional experiments, we shifted the genetic background of *Ngl3*^*−/−*^ mice from pure C57BL/6J to a hybrid 129Sv;C57BL/6J (50:50) genetic background (termed *Ngl3*^*−/−*(Hyb)^ hereafter). The two original mouse lines (C57BL/6J and 129Sv) were maintained independently before crossing and producing mice for experiments. We found that these hybrid *Ngl3*^*−/−*(Hyb)^ mice displayed normal birth rates, based on Mendelian ratios, and normal postnatal growth and survival. However, brain weight measurements and HE staining showed that the overall size of the brains from these mice was slightly reduced, although body weights were also decreased (**Fig 1D and 1E**).

An analysis of microtubule associated protein 2 (MAP2)- and Nissl-stained *Ngl3*^*−/−*^ slices revealed that the gross morphology of the *Ngl3*^*−/−*(Hyb)^ brain was largely normal, except for an apparent decrease in the size of the thalamus (**Fig 1F and 1G**). Levels of NGL-3 relatives (NGL-1 and NGL-2) were normal in the *Ngl3*^*−/−*(Hyb)^ brains (3 and 10 weeks), although levels of PTPδ, a *trans*-synaptic partner of NGL-3, were reduced (**S2 Fig**). Lastly, we examined the NGL-3 expression pattern in *Ngl3*^*−/−*(Hyb)^ mice. NGL-3 protein expression, revealed by monitoring NGL-3–β-galactosidase fusion proteins using X-gal staining (**S1 Fig**), was detected in various brain regions, including the neocortex, hippocampus, striatum, thalamus, hypothalamus, and cerebellum (**Fig 1H, S3A Fig**).

### Normal synapse development and basal excitatory transmission in the *Ngl3*^−/−(Hyb)^ hippocampus

**Fig 2:**
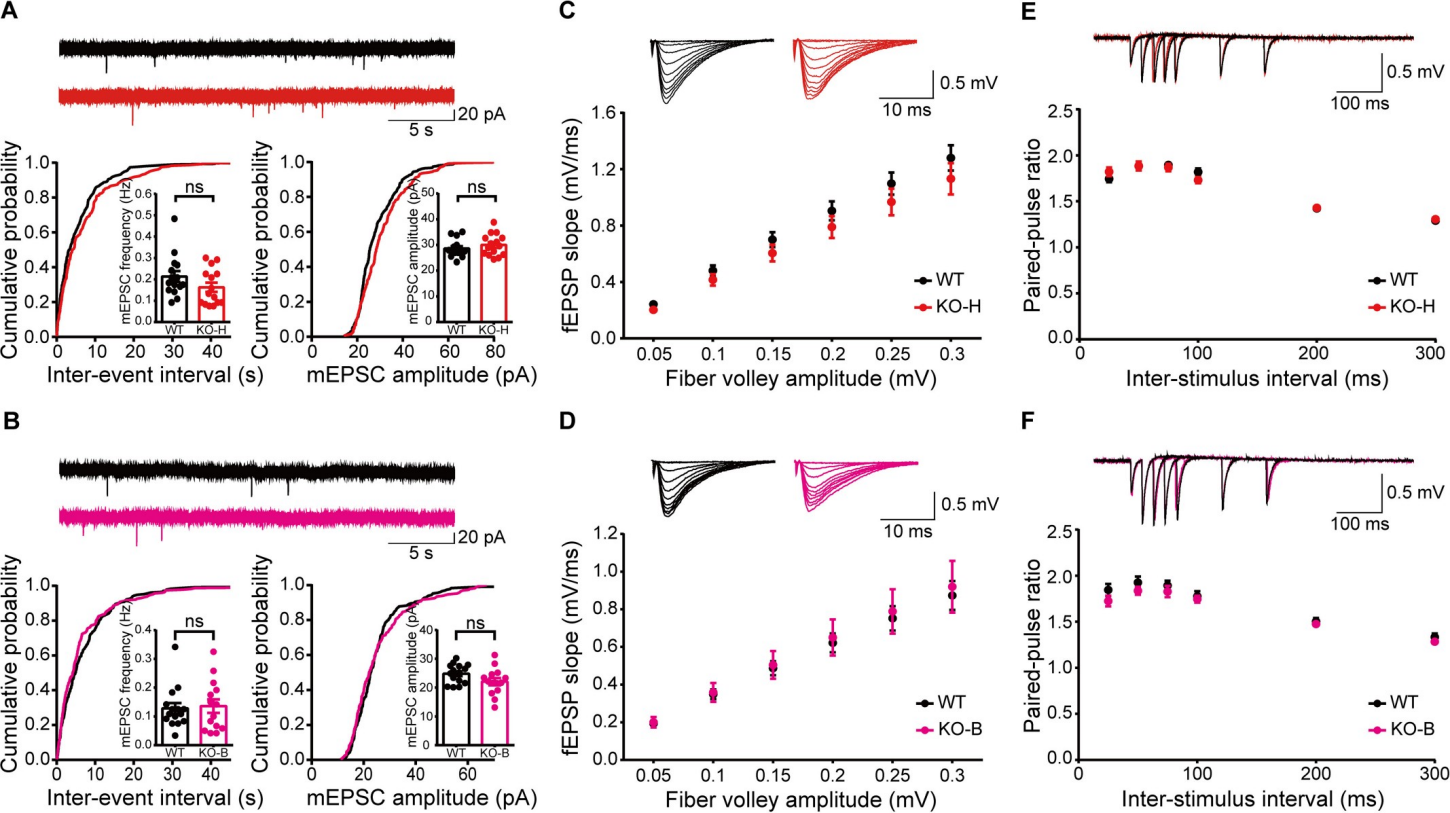
*Ngl3*^−/−(Hyb)^ and *Ngl3*^−/−(B6)^ mice show normal excitatory spontaneous and basal synaptic transmission. (A) Normal AMPAR mEPSCs in hippocampal CA1 pyramidal neurons of *Ngl3*^*−/−*(Hyb)^ mice (P21–23). *n* = 15 cells from three mice for WT and KO; ns, not significant, Student *t* test. (B) Normal AMPAR mEPSCs in hippocampal CA1 pyramidal neurons of *Ngl3*^*−/−*(B6)^ mice (P22–25). *n* = 15 cells from three mice for WT and 14, 4 for KO; ns, not significant, Student *t* test. (C and E) Normal input-output relationship and paired-pulse ratio at hippocampal SC-CA1 synapses of *Ngl3*^*−/−*(Hyb)^ mice (P28–30), as shown by fEPSP slopes plotted against either fiber volley amplitudes or inter-pulse intervals. *n* = 10 slices from three mice for WT and KO for both input-output and paired-pulse ratio, two-way ANOVA with Bonferroni test. (D and F) Normal input-output relationship and paired-pulse ratio at hippocampal SC-CA1 synapses of *Ngl3*^*−/−*(B6)^ mice (P28–30), as shown by fEPSP slopes plotted against either fiber volley amplitudes or inter-pulse intervals. *n* = 7 slices from two mice for WT and KO for both input-output and paired-pulse ratio, two-way ANOVA with Bonferroni test. Primary data can be found in S3 Data. AMPAR, AMPA receptor; CA1, Cornu Ammonis 1; fEPSP, field excitatory postsynaptic potential; KO, knockout; KO-B, knockout, C57BL/6; KO-H, knockout, hybrid; mEPSC, miniature excitatory postsynaptic current; ns, not significant; P, postnatal day; SC-CA1, Schaffer collateral-CA1 pyramidal; WT, wild-type.

To determine whether NGL-3 regulates synapse development and function in vivo, we first measured synaptic transmission in the hippocampus of *Ngl3*^*−/−*(Hyb)^ and *Ngl3*^*−/−*(B6)^ mice. Both excitatory and inhibitory synaptic transmissions were normal in *Ngl3*^*−/−*(Hyb)^ Cornu Ammonis 1 (CA1) pyramidal neurons, as shown by the frequency and amplitude of miniature excitatory postsynaptic currents (mEPSCs) and miniature inhibitory postsynaptic currents (mIPSCs), mainly mediated by AMPARs and GABA receptors, respectively (**Fig 2A; S4A Fig**). *Ngl3*^*−/−*(B6)^ CA1 pyramidal neurons also showed normal mEPSCs (**Fig 2B**). In line with these results, immunofluorescence signals for the excitatory and inhibitory presynaptic markers vesicular glutamate transporter 1(VGlut1) and vesicular GABA transporter (VGAT), respectively, were largely normal in subregions of the *Ngl3*^*−/−*(Hyb)^ hippocampus (**S3B Fig**).

In field recordings, *Ngl3*^−/−(Hyb)^ and *Ngl3*^−/−(B6)^ Schaffer collateral-CA1 pyramidal (SC-CA1) synapses showed a normal input-output relationship, which is mainly mediated by AMPARs, and paired-pulse facilitation (**Fig 2C–2F**), suggestive of unaltered basal excitatory transmission and presynaptic release probability. These results collectively suggest that *Ngl3* KO has minimal effects on excitatory synapse development and AMPAR-mediated excitatory synaptic transmission in the hippocampus.

### *Ngl3* KO moderately suppresses NMDAR-dependent synaptic transmission and long-term potentiation but near-completely suppresses LTD in the hippocampus

**Fig 3:**
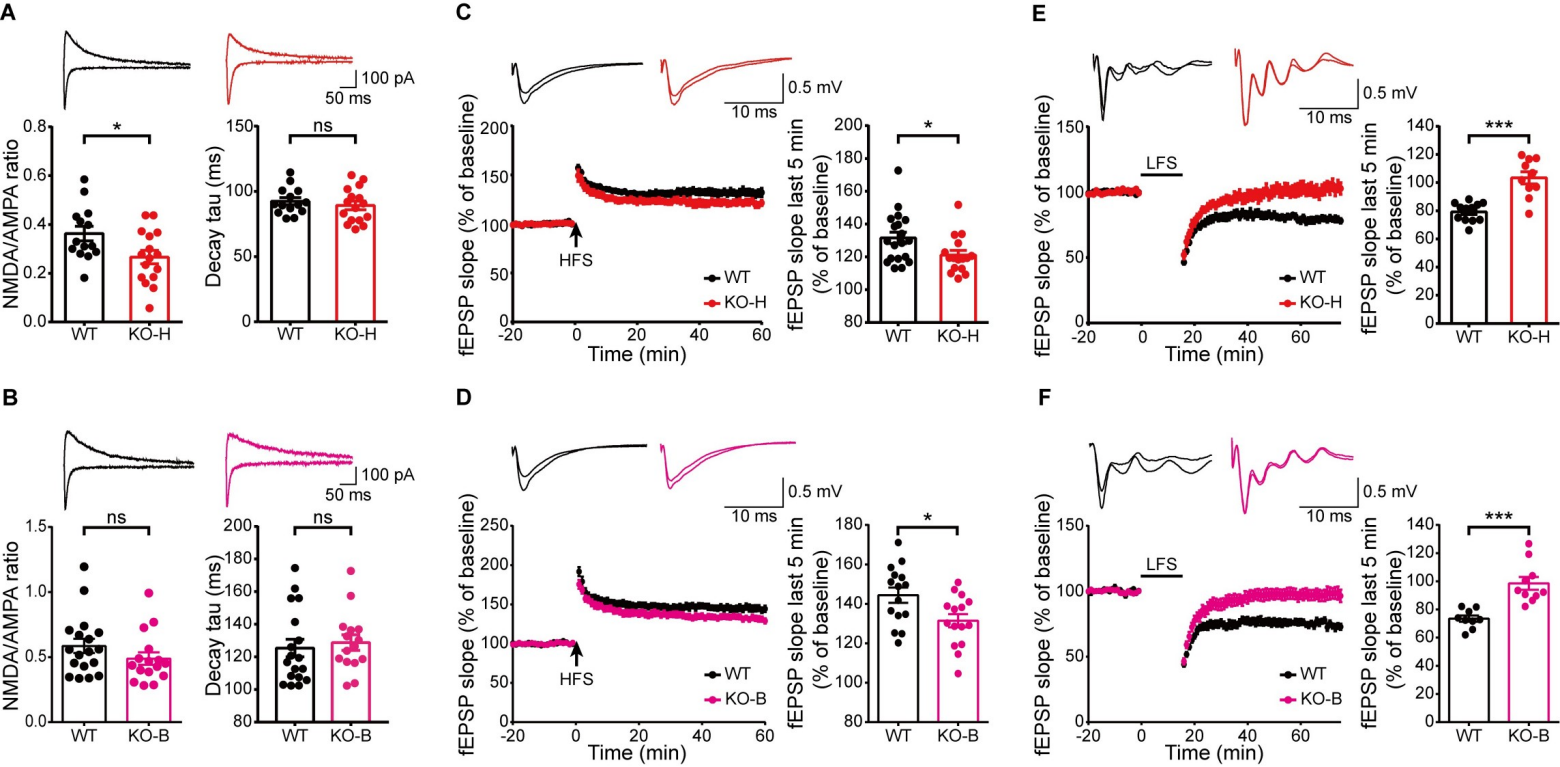
Moderate suppression of NMDAR-dependent synaptic transmission and LTP but near-complete suppression of LTD in the *Ngl3*^−/−^ hippocampus. (A) Reduced NMDAR function at hippocampal SC-CA1 synapses of *Ngl3*^−/−(Hyb)^ mice (P19–22), as shown by the NMDA/AMPA EPSC ratio. Note that the decay kinetics of NMDAR currents are normal. *n* = 14 cells from seven mice for WT and 16, 9 for KO; *P < 0.05, ns, not significant, Student *t* test. (B) Normal NMDAR function at *Ngl3*^−/−(B6)^ hippocampal SC-CA1 synapses (P19–23), as shown by the NMDA/AMPA ratio. *n* = 18 cells from eight mice for WT and 16, 10 for KO; ns, not significant, Student *t* test. (C) Suppressed LTP induced by high-frequency stimulation (100 Hz, 1 second) at hippocampal SC-CA1 synapses of *Ngl3*^−/−(Hyb)^ mice (P25–33). *n* = 19 slices from 11 mice for WT and 16, 8 for KO; *P < 0.05, Student *t* test. (D) Suppressed LTP induced by high-frequency stimulation (100 Hz, 1 second) at hippocampal SC-CA1 synapses of *Ngl3*^−/−(B6)^ mice (P23–26). *n* = 15 slices from nine mice for WT and 15, 10 for KO; *P < 0.05, Student *t* test. (E) Near-complete suppression of LTD induced by low-frequency stimulation (1 Hz, 15 minutes) at hippocampal SC-CA1 synapses of *Ngl3*^−/−(Hyb)^ mice (P16–20). *n* = 12, 6 for WT and 10, 6 for KO; ***P < 0.001, Student *t* test. (F) Near-complete suppression of LTD induced by low-frequency stimulation (1 Hz, 15 minutes) at hippocampal SC-CA1 synapses of *Ngl3*^−/−(B6)^ mice (P17–21). *n* = 9, 6 for WT and 10, 6 for KO; ***P < 0.001, Student *t* test. Primary data can be found in S3 Data. AMPA, α-amino-3-hydroxy-5-methyl-4-isoxazolepropionic acid; EPSC, excitatory postsynaptic current; fEPSP, field excitatory postsynaptic potential; HFS, high-frequency stimulation; KO, knockout; KO-B, knockout, C57BL/6; KO-H, knockout, hybrid; LTD, long-term depression; LTP, long-term potentiation; NMDA, N-methyl-D-aspartate; NMDAR, NMDA receptor; ns, not significant; P, postnatal day; SC-CA1, Schaffer collateral-CA1 pyramidal; WT, wild-type.

Because artificially clustered NGL-3 has been shown to co-cluster with NMDARs in cultured hippocampal neurons [22], we next attempted to determine whether *Ngl3* KO affects NMDAR-mediated synaptic transmission and NMDAR-dependent synaptic plasticity. Patch-clamp recordings showed significantly reduced NMDAR function at *Ngl3*^−/−(Hyb)^ SC-CA1 synapses, as evidenced by the decrease in the ratio of evoked NMDAR-to-AMPAR–mediated excitatory postsynaptic currents (EPSCs) (NMDA/AMPA ratio) (**Fig 3A**). *Ngl3*^−/−(B6)^ SC-CA1 synapses, however, did not show a change in the NMDA/AMPA ratio (**Fig 3B**), likely attributable to the differential impacts of *Ngl3* KO on brain development under different genetic backgrounds. In addition, NMDAR-dependent mEPSCs from *Ngl3*^−/−(Hyb)^ mice measured in the presence of AMPAR blocker 2,3-dihydroxy-6-nitro-7-sulfamoyl-benzo(F)quinoxaline (NBQX) and under a low-magnesium condition indicated decreases in the amplitude and frequency of NMDAR-dependent mEPSCs (**S4B Fig**). The decreased frequency of NMDAR mEPSCs, given the normal frequency of AMPAR mEPSCs (**Fig 2A**), appears to be caused by the reduced amplitude of NMDAR mEPSCs, which makes peak sizes smaller than the detection threshold. The normal input/output curve of evoked EPSCs and normal AMPAR mEPSCs, together with the suppressed NMDAR mEPSCs, in the *Ngl3*^−/−(Hyb)^ hippocampus strongly suggest a selective decrease in NMDAR currents.

In line with the moderately reduced NMDAR function, long-term potentiation (LTP) induced by high-frequency stimulation (HFS) was significantly suppressed (33%–40%) at *Ngl3*^−/−(Hyb)^ and *Ngl3*^−/−(B6)^ SC-CA1 synapses (**Fig 3C and 3D**). Intriguingly, LTD induced by low-frequency stimulation (LFS) was almost completely abolished at both *Ngl3*^−/−(Hyb)^ and *Ngl3*^−/−(B6)^ SC-CA1 synapses (**Fig 3E and 3F**). In contrast, metabotropic glutamate receptor (mGluR)-dependent LTD was not affected at *Ngl3*^−/−(Hyb)^ SC-CA1 synapses (**S4C Fig**). These results collectively suggest that *Ngl3* KO leads to moderate suppression of NMDAR-dependent synaptic transmission and LTP but near-complete suppression of LTD in the hippocampus.

### *Ngl3* KO does not affect synaptic or surface levels of NMDARs in the brain but strongly activates Akt and inhibits GSK3𝛃

**Fig 4:**
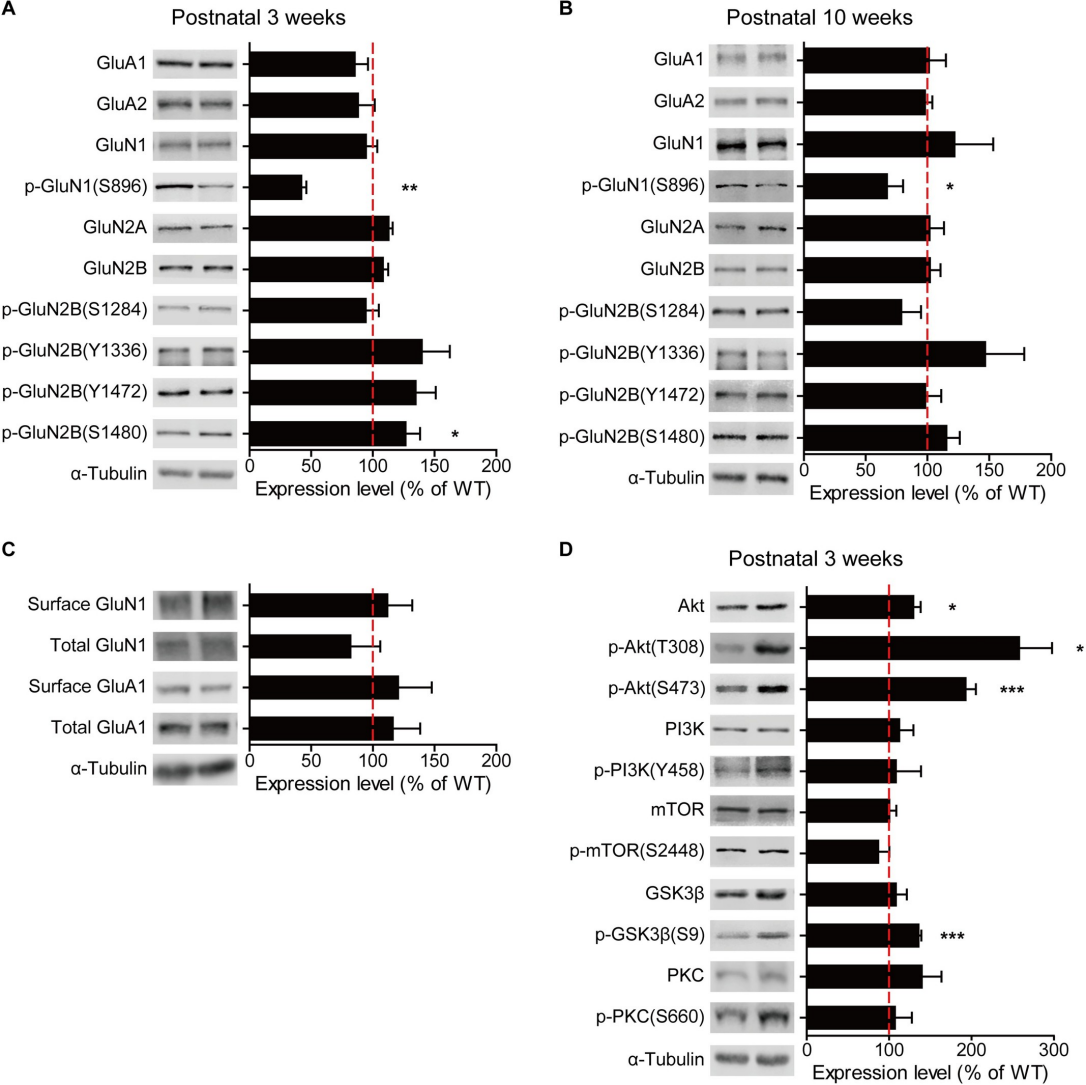
*Ngl3* KO does not affect synaptic or surface levels of NMDARs in the brain but strongly activates Akt and inhibits GSK3β. (A and B) *Ngl3* KO does not affect total and phosphorylation levels of NMDAR subunits, as shown by immunoblot analyses with whole-brain crude synaptosomes from *Ngl3*^−/−(Hyb)^ mice (3 and 10 weeks). KO levels of the indicated proteins normalized to α-tubulin levels were further normalized to WT levels normalized to α-tubulin. *n* = 4 mice for WT and KO. (C) *Ngl3* KO does not affect surface levels of GluN1 or GluA1, as shown by biotinylated surface levels of the indicated proteins from the total lysates of the *Ngl3*^−/−(Hyb)^ hippocampus (3 weeks). *n* = 5 mice for WT and 6 mice for KO. (D) *Ngl3* KO leads to strong increases in the phosphorylation levels of Akt (Ser-308 and Ser-473) and GSK3β (Ser-9), as shown by the immunoblot analysis of the indicated proteins with whole-brain crude synaptosomes of *Ngl3*^−/−(Hyb)^ mice (3 weeks). *n* = 4 mice for WT and KO (except for *n* = 3 and 4 for pAkt-Ser-308). Primary data can be found in S3 Data. GluA1, Glutamate, AMPAR subunit 1; GluA2, Glutamate, AMPAR subunit 2; GluN1, Glutamate, NMDAR subunit 1; GSK3β, glycogen synthase kinase 3β; KO, knockout; mTOR, mammalian target of rapamycin; NMDAR, NMDA receptor; pAkt, phosphorylated Akt; p-GluN1, phosphorylated GluN1; p-GluN2B, phosphorylated GluN2B; p-GSK3β, phosphorylated GSK3β; PKC, protein kinase C; p-PKC, phosphorylated PKC; p-PI3K, phosphorylated PI3K; PI3K, phosphatidyl inositol 3 kinase; WT, wild-type.

In order to explore the mechanisms underlying the moderate decreases in NMDAR-mediated synaptic transmission and LTP and the near-complete elimination of LTD in *Ngl3*^−/−(Hyb)^ mice (**Fig 3**), we tested whether there are any changes in the synaptic levels of NMDAR subunits in the *Ngl3*^−/−^ brain. However, *Ngl3* KO did not affect the synaptic levels of NMDAR subunits, as shown by the immunoblot analysis of Glutamate, NMDAR subunit 1 (GluN1), Glutamate, NMDAR subunit 2A (GluN2A), and Glutamate, NMDAR subunit 2B (GluN2B) in the crude synaptosomal fraction of WT and *Ngl3*^−/−(Hyb)^ whole brains at both 3 and 10 postnatal weeks (**Fig 4A and 4B**). In addition, *Ngl3* KO did not affect the surface levels of the GluN1 subunit of NMDARs (**Fig 4C**), suggesting that surface trafficking of NMDARs was not affected.

The lack of changes in the synaptic and surface levels of NMDAR subunits in the *Ngl3*^−/−(Hyb)^ brain (**Fig 4**) suggests the possibility that *Ngl3* KO may affect NMDAR-dependent synaptic transmission and synaptic plasticity (LTP and LTD) by altering the phosphorylation levels of NMDARs or the signaling pathways in the downstream of NMDAR activation [32]. To this end, we first measured phosphorylation levels of NMDAR subunits at known sites and found a significant decrease in the phosphorylation of GluN1 at Ser-896 but not in GluN2B at Ser-1284, Tyr-1336, Tyr-1472, or Ser-1480 in the *Ngl3*^−/−(Hyb)^ brain (**Fig 4A and 4B**). Given that the GluN1 phosphorylation at Ser-896 promotes the surface trafficking of GluN1 from the endoplasmic reticulum [33] but that our *Ngl3* KO does not lead to a decrease in surface levels of GluN1 (**Fig 4C**), a compensatory change, such as decreased endocytosis of NMDARs, might have occurred to normalize the surface levels of NMDARs in the mutant neurons.

We next measured changes in the total and phosphorylation levels of known signaling molecules in the *Ngl3*^−/−(Hyb)^ brain (3 weeks). We found a strong increase in the phosphorylation levels of the serine/threonine kinase Akt (also known as protein kinase B) at both Ser-308 and Ser-473 (**Fig 4D**), known to lie in the downstream of phosphatidyl inositol 3 kinase/3-phosphoinositide-dependent protein kinase 1 (PI3K/PDK1) and mammalian target of rapamycin (mTOR) complex 2 (mTORC2), respectively [34]. Total Akt levels were modestly increased. Unexpectedly, there were no changes in the total and phosphorylation levels of PI3K (Tyr-458) and mTOR (Ser-2448), which lie in the upstream and downstream of Akt, respectively, suggesting that the observed increase in Akt activity may not involve the canonical PI3K-Akt-mTOR pathway or that PI3K/mTOR activity was normalized by compensation. Intriguingly, however, there was a strong increase in the phosphorylation (a measure of inactivation), but not total, levels of GSK3β at Ser-9, a serine/threonine kinase in the downstream of Akt known to promote LTD when activated (dephosphorylated) [35, 36] (**Fig 4D**). Total and phosphorylation levels of protein kinase C (PKC), known to phosphorylate and stimulate NMDAR function [37], were not affected. These results suggest that *Ngl3* KO leads to strong increases in the phosphorylation of Akt and GSK3β.

### Inhibition of Akt, but not activation of NMDAR, normalizes the suppressed LTD in the *Ngl3*^−/−(Hyb)^ hippocampus

**Fig 5:**
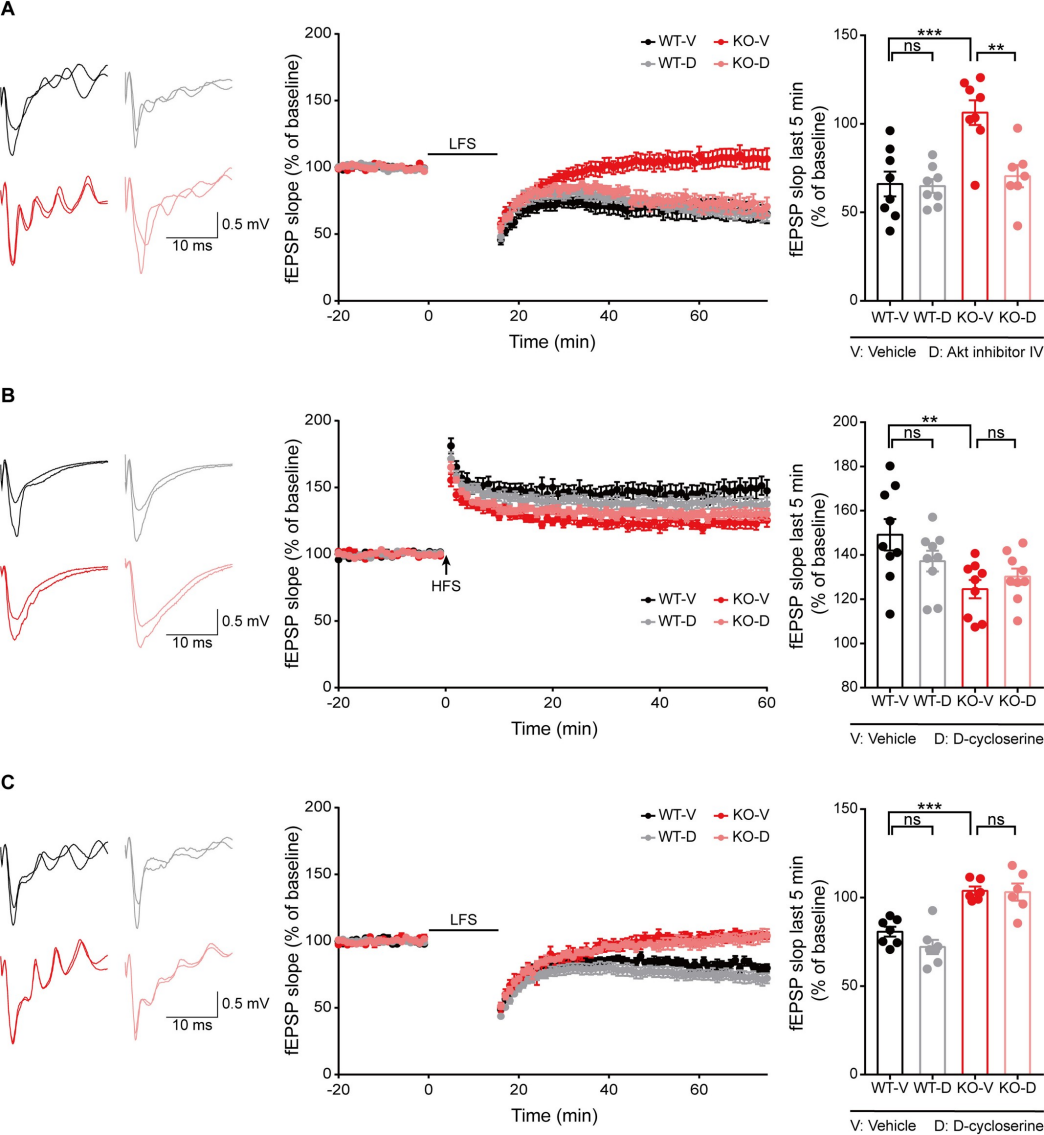
Inhibition of Akt, but not activation of NMDAR, normalizes the suppressed LTD in the *Ngl3*^−/−(Hyb)^ hippocampus. (A) Akt inhibitor IV normalizes the suppressed LTD induced by LFS (1 Hz, 15 minutes) at hippocampal SC-CA1 synapses in *Ngl3*^−/−(Hyb)^ mice (P16–20). *n* = 8 slices from three mice for WT-V, 8, 3 for WT-D, 8, 3 for KO-V, and 7, 3 for KO-D; **P < 0.01, ***P < 0.001, ns, not significant, two-way ANOVA with Bonferroni test. (B) DCS has no effect on LTP induced by HFS (100 Hz, 1 second) at hippocampal SC-CA1 synapses of *Ngl3*^−/−(Hyb)^ mice (P26–32). *n* = 9 slices from three mice for WT-V, WT-D, KO-V, and KO-D; **P < 0.01, ns, not significant, two-way ANOVA with Bonferroni test. (C) DCS has no effect on LTD induced by LFS (1 Hz, 15 minutes) at hippocampal SC-CA1 synapses of *Ngl3*^−/−(Hyb)^ mice (P16–20). *n* = 7 slices from six mice for WT-V, 7, 4 for WT-D, 6, 5 for KO-V, and 6, 4 for KO-D; ***P < 0.001, ns, not significant, two-way ANOVA with Bonferroni test. Primary data can be found in S3 Data. DCS, D-cycloserine; fEPSP, field excitatory postsynaptic potential; HFS, high-frequency stimulation; KO-D, knockout, drug; KO-V, knockout, vehicle; LFS, low-frequency stimulation; LTD, long-term depression; LTP, long-term potentiation; NMDAR, NMDA receptor; ns, not significant; P, postnatal day; SC-CA1, Schaffer collateral-CA1 pyramidal; WT-D, wild-type, drug; WT-V, wild-type, vehicle.

The strongly increased phosphorylation of Akt and GSK3β in the *Ngl3*^−/−(Hyb)^ brain (**Fig 4D**) suggests the possibility that altered Akt/GSK3β signaling may induce the near-complete elimination of LTD in the hippocampus (**Fig 3**). To this end, we measured LTD induced by LFS in at *Ngl3*^−/−(Hyb)^ hippocampal SC-CA1 synapses in the presence of the Akt inhibitor IV (10 μM) (**Fig 5A**), known to inhibit Akt phosphorylation at both Ser-308 and Ser-473 [38–40]. Surprisingly, this Akt inhibition normalized the suppressed LFS-LTD at *Ngl3*^−/−(Hyb)^ SC-CA1 synapses, as compared with vehicle-treated *Ngl3*^−/−(Hyb)^ synapses. In contrast, this Akt inhibition did not affect WT LFS-LTD. These results suggest that the enhanced Akt/GSK3β phosphorylation may suppress LTD in the *Ngl3*^−/−(Hyb)^ hippocampus.

Because NMDAR-mediated synaptic transmission and LTP were reduced in the *Ngl3*^−/−(Hyb)^ hippocampus (**Fig 3**), and reduced NMDAR function can contribute to NMDAR-dependent LTP and LTD [36, 41], we tested if the suppressed LTP and LTD could be normalized by the NMDAR agonist D-cycloserine (DCS). However, neither LTP nor LTD was normalized by DCS (20 μM) at *Ngl3*^−/−(Hyb)^ hippocampal SC-CA1 synapses (**Fig 5B and 5C**). These results suggest that the decreased NMDAR-mediated synaptic transmission does not contribute to the suppressed LTP or LTD in the *Ngl3*^−/−(Hyb)^ hippocampus.

### *Ngl3*^−/−^ mice display hyperactivity, anxiolytic-like behavior, and impaired learning and memory

*Ngl3* KO leads to the alteration of LTP and LTD (**Fig 3**), which would affect the formation and function of neural circuits in the *Ngl3*^−/−^ brain. In addition, although NGL-3 has not been directly implicated in any brain disorders, its presynaptic binding partners such as LAR-RPTPs have been strongly associated with various brain disorders [1, 4], including restless leg syndrome [42–44], ADHD [45], autism spectrum disorder (ASD) [46], and bipolar disorder [47]. We thus tested if *Ngl3* KO leads to any behavioral abnormalities in *Ngl3*^−/−(Hyb)^ and *Ngl3*^−/−(B6)^ mice.

**Fig 6:**
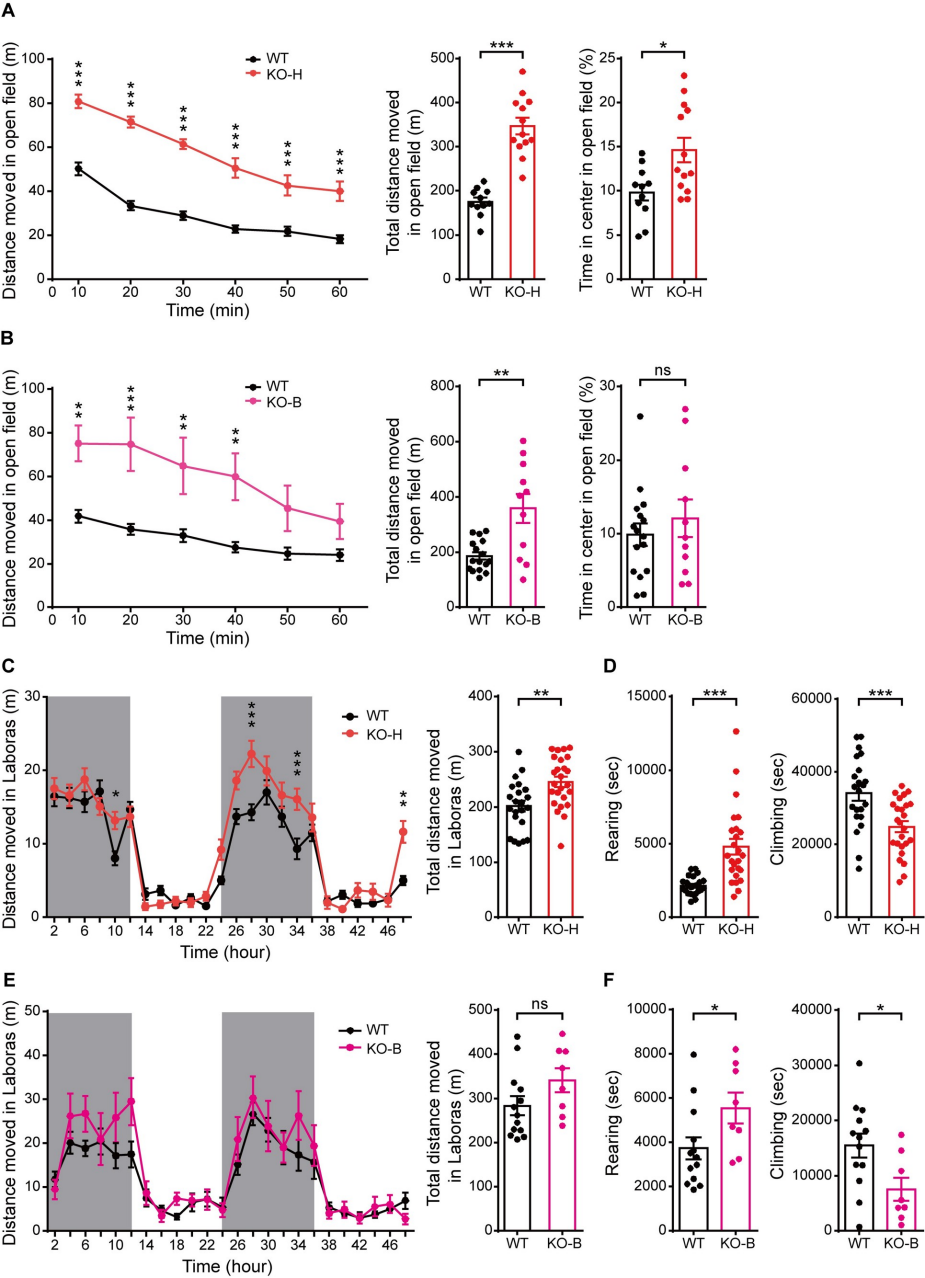
*Ngl3*^−/−(Hyb)^ and *Ngl3*^−/−(B6)^ mice display hyperactivity. (A) Hyperactivity of *Ngl3*^−/−(Hyb)^ mice (2–4 months) in the open-field test. Data are presented as means ± SEM. *n* = 11 mice for WT and 13 for KO. *P < 0.05, ***P < 0.001, two-way ANOVA with Bonferroni test and Student *t* test. (B) Hyperactivity of *Ngl3*^−/−(B6)^ mice (2–4 months) in the open-field test. Data are presented as means ± SEM. *n* = 16 mice for WT and 11 for KO. **P < 0.01, ***P < 0.001, ns, not significant, two-way ANOVA with Bonferroni test and Student *t* test. (C and D) Hyperactivity of *Ngl3*^−/−(Hyb)^ mice (2–4 months) in the Laboras test, in which mouse movements are continuously monitored for 48 hours. Note that rearing, but not climbing, is increased in Laboras cages, likely reflecting vertical hyperactivity. The 12-hour shades indicate light-off periods. *n* = 22 mice for WT and 25 for KO, *P < 0.05, **P < 0.01, ***P < 0.001, two-way ANOVA with Bonferroni test and Student *t* test. (E and F) Normal locomotor activity of *Ngl3*^*−/−*(B6)^ mice (2–4 months) in the Laboras test, in which mouse movements are continuously monitored for 48 hours. Note that rearing, but not climbing, is increased in Laboras cages, likely reflecting vertical hyperactivity. *n* = 13 mice for WT and 8 for KO, *P < 0.05, ns, not significant, two-way ANOVA and Student *t* test. Primary data can be found in S3 Data. KO, knockout; KO-B, knockout, C57BL/6; KO-H, knockout, hybrid; ns, not significant; WT, wild-type.

*Ngl3*^−/−(Hyb)^ mice showed strong hyperactivity in the open-field test and spent more time in the center region of the open-field arena compared with WT mice (**Fig 6A**). *Ngl3*^−/−(B6)^ mice showed similar open-field hyperactivity but did not display a change in the center time (**Fig 6B**).

Mouse movements were then monitored in a home-cage–like environment (Laboras cages), which becomes a familiar environment after a few hours of initial habituation, for two consecutive days. Under these conditions, *Ngl3*^−/−(Hyb)^ mice also showed strong hyperactivity, especially on the second day during the light-off period (**Fig 6C**). In addition, they showed increased rearing, a measure of vertical hyperactivity, but exhibited reduced climbing activity (**Fig 6D**). *Ngl3*^−/−(B6)^ mice showed a similar increase in rearing and a decrease in climbing but no detectable hyperactivity in Laboras cages (**Fig 6E and 6F**). These results collectively suggest that *Ngl3* KO leads to hyperactivity in both novel and familiar environments.

**Fig 7:**
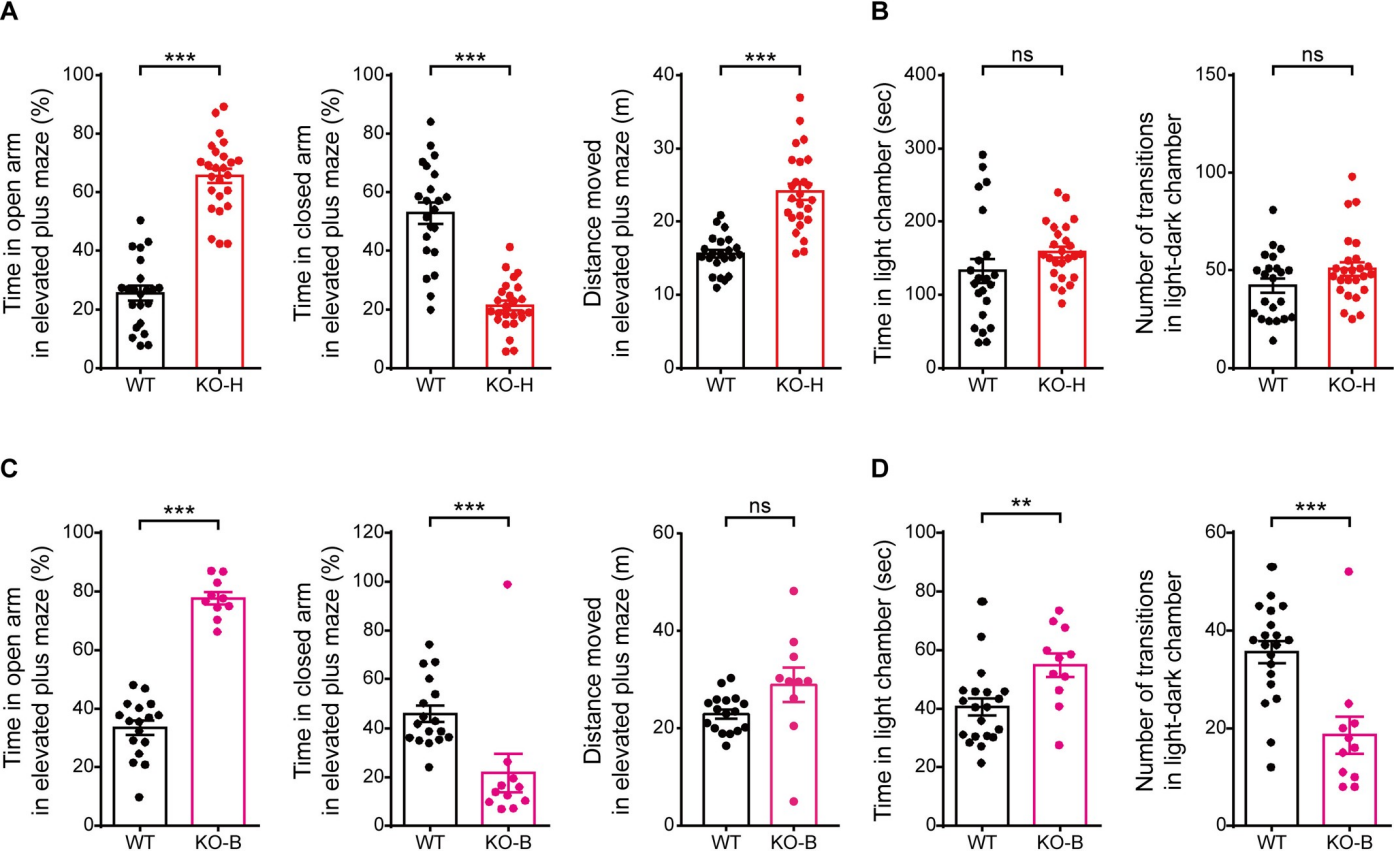
*Ngl3*^−/−(Hyb)^ and *Ngl3*^−/−(B6)^ mice display anxiolytic-like behavior. (A) Anxiolytic-like behavior of *Ngl3*^−/−(Hyb)^ mice (2–4 months) in the elevated plus maze test, as shown by time spent in open/closed arms. Note that *Ngl3*^−/−(Hyb)^ mice are also hyperactive in this test. *n* = 22 mice for WT and 25 for KO, ***P < 0.001, Student *t* test. (B) Normal anxiety-related behavior of *Ngl3*^−/−(Hyb)^ mice (2–4 months) in the light-dark test, as shown by transition number and chamber time. *n* = 22 mice for WT and 25 for KO; ns, not significant, Student *t* test. (C) Anxiolytic-like behavior of *Ngl3*^−/−(B6)^ mice (2–4 months) in the elevated plus maze test, as shown by time spent in open/closed arms. *n* = 17 mice for WT and 10 for KO; ***P < 0.001, ns, not significant, Student *t* test. (D) Anxiolytic-like behavior of *Ngl3*^−/−(B6)^ mice (2–4 months) in the light-dark test, as shown by time in the light chamber and transition number. *n* = 20 mice for WT and 11 for KO; **P < 0.01, ***P < 0.001, Student *t* test. Primary data can be found in S3 Data. KO, knockout; KO-B, knockout, C57BL/6; KO-H, knockout, hybrid; ns, not significant; WT, wild-type.

*Ngl3*^−/−(Hyb)^ mice were also hyperactive on the elevated plus maze (**Fig 7A**), similar to their behavior in the open-field arena. In addition, *Ngl3*^−/−(Hyb)^ mice showed reduced anxiety-like behavior, as evidenced by the increased time spent in open arms and decreased time spent in closed arms in the elevated plus maze (**Fig 7A**). This anxiolytic-like behavior in the elevated plus maze is consistent with the increased time spent in the center region of the open-field arena (**Fig 6A**). However, *Ngl3*^−/−^ mice acted normally in the light-dark chamber test (**Fig 7B**), suggesting that their anxiolytic-like behaviors manifest under specific conditions. *Ngl3*^−/−(B6)^ mice were less anxious on the elevated plus maze (**Fig 7C**) as well as in the light-dark apparatus (**Fig 7D**), displaying stronger anxiolytic-like phenotypes as compared with *Ngl3*^−/−(Hyb)^ mice. These results collectively suggest that *Ngl3* KO leads to anxiolytic-like behaviors in mice.

An analysis of *Ngl3*^−/−(Hyb)^ mice for autistic-like behaviors revealed normal social interactions in the three-chamber test (**S5A Fig**). These mice also showed normal repetitive behaviors, including self-grooming and marble burying (**S5B Fig**). *Ngl3*^−/−(B6)^ mice also acted normally in the three-chamber test (**S5C Fig**).

**Fig 8:**
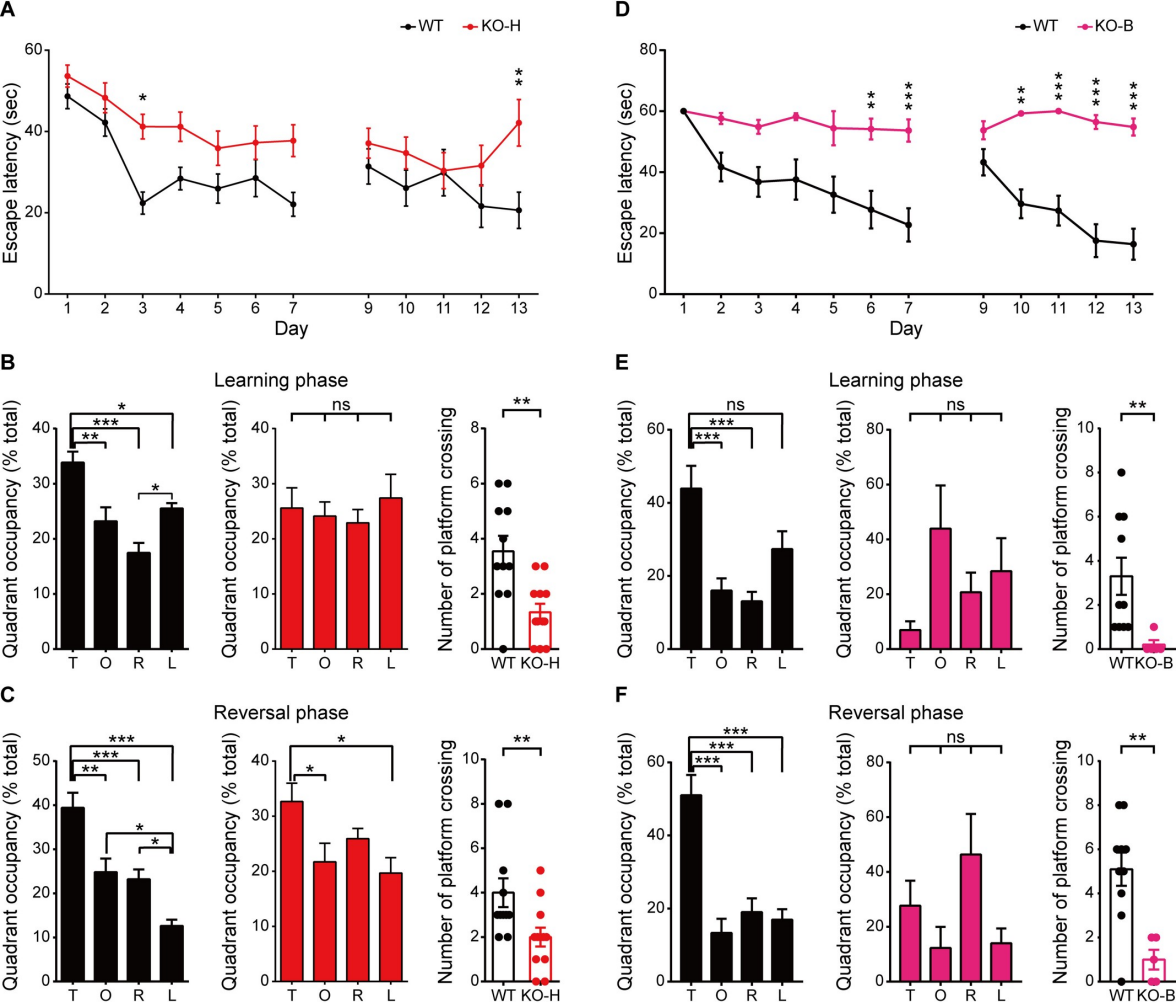
*Ngl3*^−/−(Hyb)^ and *Ngl3*^−/−(B6)^ mice display impaired learning and memory. (A–C) *Ngl3*^−/−(Hyb)^ mice (2–4 months) display impaired spatial learning and memory in learning (days 1–7), probe (days 8 and 14), and reversal (days 9–13) phases of the Morris water maze test (days 1–14). Quadrant occupancy and number of crossings over the location of the former platform during the probe phase on day 8 (B) and on day 14 after the completion of reversal learning (C) are also indicated. *n* = 11 mice for WT and 12 for KO; *P < 0.05, **P < 0.01, ***P < 0.001, ns, not significant, repeated measure of ANOVA, Student *t* test, and Mann-Whitney test. (D–F) *Ngl3*^−/−(B6)^ mice (2–4 months) display impaired spatial learning and memory in learning (days 1–7), probe (days 8 and 14), and reversal (days 9–13) phases of the Morris water maze test (days 1–14). Quadrant occupancy and number of crossings over the location of the former platform are indicated for the probe phases on day 8 after the completion of the initial learning phase (B) and on day 14 after the completion of the reversal learning phase (C). *n* = 10 mice for WT and 5 for KO; **P < 0.01, ***P < 0.001, ns, not significant, repeated measure of ANOVA and Student *t* test. Primary data can be found in S3 Data. KO, knockout; KO-B, knockout, C57BL/6; KO-H, knockout, hybrid; L, left; ns, not significant; O, opposite; R, right; T, target quadrant; WT, wild-type.

Given that *Ngl3*^−/−^ mice show altered synaptic plasticity in the hippocampus (**Fig 3**), we next examined learning and memory in these mice. *Ngl3*^−/−(Hyb)^ mice showed impaired learning and memory in the Morris water maze test during learning, probe, and reversal phases (**Fig 8A–8C**). Similar impairments were observed in *Ngl3*^−/−(B6)^ mice (**Fig 8D–8F**). In contrast, *Ngl3*^−/−(Hyb)^ mice showed normal memory in novel-object recognition and fear conditioning tests (**S5D and S5E Fig**). *Ngl3*^−/−(B6)^ mice also performed normally in the novel-object recognition test (**S5F Fig**). *Ngl3*^−/−(Hyb)^ mice showed impaired motor coordination and learning in the rotarod test (**S5G Fig**). These results suggest that *Ngl3* KO leads to deficits in specific learning and memory behaviors.

### *Ngl3* heterozygosity minimally affects synaptic functions and behaviors in mice

To determine whether *Ngl3* deletion has dose-dependent impacts, we first tested if *Ngl3* heterozygosity affects synaptic transmission and plasticity. We used brain slices from *Ngl3*^+/–(B6)^ mice, which are likely to display stronger synaptic phenotypes relative to *Ngl3*^+/–(Hyb)^ mice based on their stronger neurodevelopmental phenotypes. Heterozygous *Ngl3*^+/–(B6)^ mice, however, showed normal spontaneous and basal synaptic transmission in the hippocampus, as shown by mEPSCs and mIPSCs in CA1 pyramidal neurons and input-output and paired-pulse ratios at SC-CA1 synapses (**S6A–S6D Fig**). NMDAR function was also normal, as measured by the NMDA/AMPA ratio and LTP induced by HFS at *Ngl3*^+/–^ SC-CA1 synapses, although these parameters tended to decrease (**S6E and S6F Fig**).

Behaviors of *Ngl3*^+/–(B6)^ mice were largely normal, although these mice showed moderate hypoactivity (not hyperactivity) in the open-field test and moderate anxiolytic-like behavior in the light-dark test (**S7 Fig**). These results suggest that heterozygosity of *Ngl3* has minimal effects on synaptic transmission and plasticity and behaviors in mice.

### Enhanced hippocampal excitation and susceptibility to induced seizures in *Ngl3*^−/−(Hyb)^ mice

Altered NMDAR function and synaptic plasticity (LTP and LTD) at *Ngl3*^−/−^excitatory synapses would disrupt the balance between excitation and inhibition at the synapse, neuron, and circuit levels, known to be required for normal brain functions and behaviors [48–51].

To this end, we first tested whether *Ngl3* is expressed in inhibitory GABAergic neurons in addition to excitatory glutamatergic neurons by double fluorescence in situ hybridization. *Ngl3* mRNAs were detected in both excitatory and inhibitory neurons in brain regions, including the cortex and hippocampus (**S8 Fig**). These results suggest the possibility that NGL-3 regulates excitatory synapse development and function in inhibitory neurons and the balance between excitatory and inhibitory neurons.

**Fig 9:**
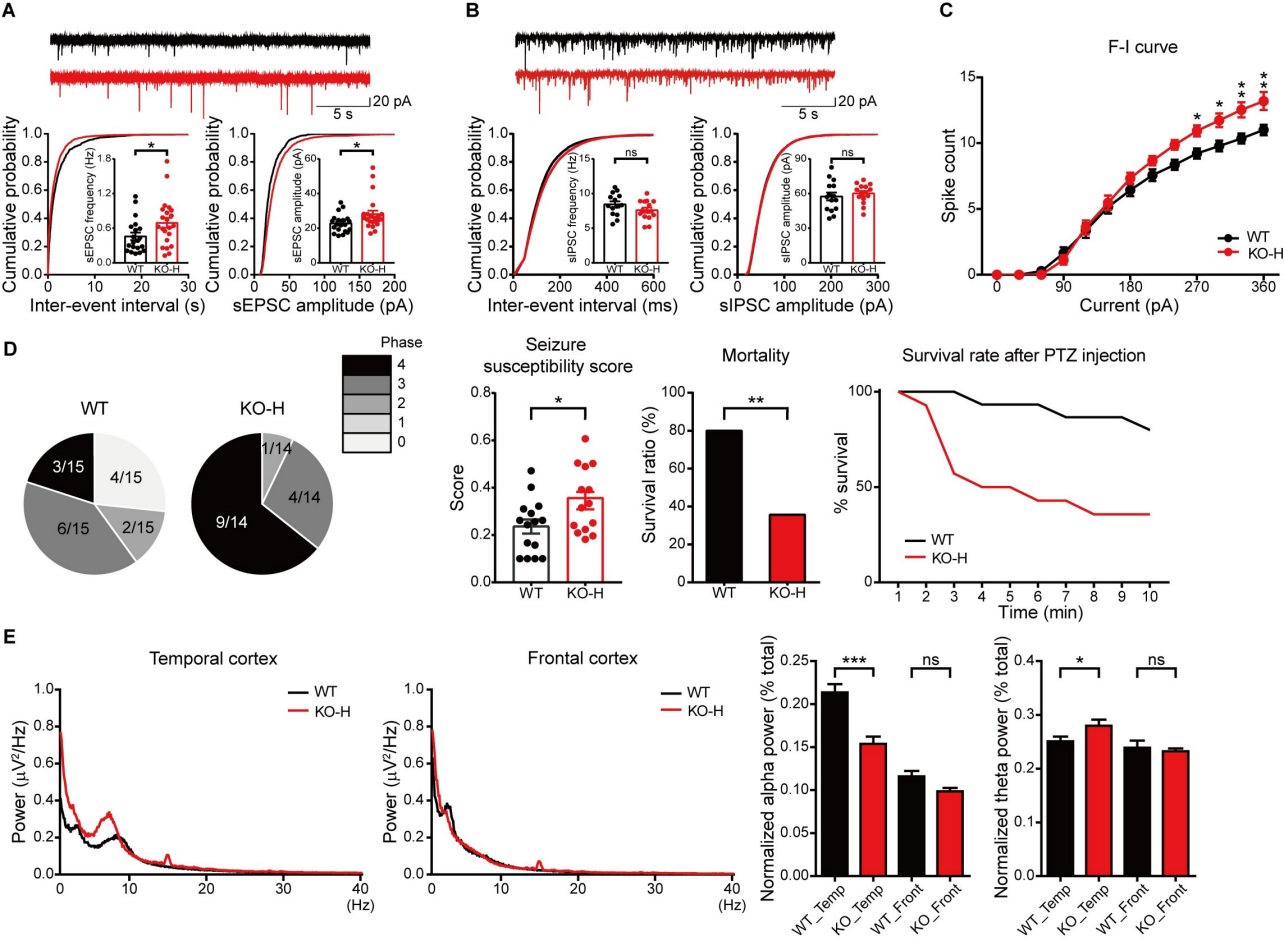
Enhanced hippocampal excitation, susceptibility to induced seizures, and altered brain oscillations in *Ngl3*^−/−(Hyb)^ mice. (A and B) Increased frequency and amplitude of sEPSCs, but normal sIPSCs, in *Ngl3*^−/−(Hyb)^ hippocampal CA1 pyramidal neurons (P23–25 for sEPSCs and P22–23 for sIPSCs). *n* = 20 cells from six mice for WT and 22, 6 for KO (sEPSC); 15, 4 for WT and 15, 3 for KO (sIPSC); *P < 0.05, ns, not significant, Student *t* test. (C) Increased excitability of *Ngl3*^−/−(Hyb)^ hippocampal CA1 neurons (P21–23), as shown by spike counts in response to current injections. *n* = 14 cells from three mice for WT and KO; *P < 0.05, **P < 0.01, two-way ANOVA with Bonferroni test. (D) Enhanced susceptibility to PTZ-induced seizures in *Ngl3*^−/−(Hyb)^ mice (3 months), as shown by seizure susceptibility score and mortality. *n* = 15 mice for WT and 14 for KO; *P < 0.05, **P < 0.01, Student *t* test. (E) Decreased alpha and increased theta oscillations in the temporal, but not frontal, lobes of the *Ngl3*^−/−(Hyb)^ brain (3 months), as shown by the power spectrum and normalized power in different frequency ranges. Frequency ranges in Hz were defined as follows: delta, 1–4; theta, 4–8; alpha, 8–13; beta, 13–30; gamma, 30–100. *n* = 6 mice for WT and KO; *P < 0.05, ***P < 0.001, ns, not significant, Student *t* test. Primary data can be found in S3 Data. CA1, Cornu Ammonis 1; F-I curve, firing-I (current) curve; KO, knockout; KO-H, knockout, hybrid; ns, not significant; P, postnatal day; PTZ, pentylenetetrazol; sEPSC, spontaneous EPSC; sIPSC, spontaneous IPSC; WT, wild-type.

To further explore this possibility, we next examined network activities in the *Ngl3*^−/−(Hyb)^ hippocampus by measuring spontaneous EPSCs (sEPSCs) and sIPSCs in CA1 pyramidal neurons. This analysis revealed increases in the frequency and amplitude of sEPSCs, although the latter was increased to a lesser extent (**Fig 9A**); by contrast, the frequency and amplitude of mEPSCs were normal in the same neuronal population (**Fig 2A**). sIPSCs were normal in *Ngl3*^−/−(Hyb)^ CA1 pyramidal neurons (**Fig 9B**), similar to the mIPSC results (**S4A Fig**). These results suggest that *Ngl3* deletion does not affect inhibitory synaptic input in the hippocampus but increases the excitatory drive by enhancing excitatory synaptic input.

The increased excitatory drive in the hippocampus could be attributable to enhanced excitatory inputs from outside the hippocampus but could also involve increased neuronal excitability within the hippocampus. Intriguingly, neuronal excitability was significantly increased in *Ngl3*^−/−(Hyb)^ CA1 pyramidal neurons, measured as spike counts induced by injected currents (**Fig 9C**). These results suggest that *Ngl3* KO leads to enhanced excitatory drive in the hippocampus.

To test whether the abovementioned changes are associated with any changes in the excitatory drive in the whole brain, we induced seizures in *Ngl3*^−/−(Hyb)^ mice by intraperitoneally injecting pentylenetetrazol (PTZ) (40 mg/kg). These experiments showed that both seizure-susceptibility scores and mortality were increased in PTZ-injected *Ngl3*^−/−(Hyb)^ mice (**Fig 9D**). Because the enhanced excitatory drive might be associated with altered brain oscillations, we measured rhythmic oscillations in the frontal and temporal lobes of the *Ngl3*^−/−(Hyb)^ brain and found decreased alpha oscillations and increased theta oscillations in the temporal, but not frontal lobes (**Fig 9E, S9 Fig)**. These results suggest that *Ngl3* KO in mice induces an increased susceptibility to induced seizures that is associated with oscillatory abnormalities.

### NMDAR activation rescues hyperactivity and NMDAR currents, but not anxiolytic-like behavior, in *Ngl3*^−/−(Hyb)^ mice

The reduced NMDAR-mediated synaptic transmission does not seem to suppress LTP and LTD in *Ngl3*^−/−(Hyb)^ mice, as shown by the lack of the rescue effect of DCS on LTP and LTD (**Fig 5B and 5C**). However, the reduced NMDAR currents could still suppress the excitability of dendrites and neuronal output function [52–54], disrupting normal behaviors in *Ngl3*^−/−(Hyb)^ mice. To this end, we tested whether DCS could improve the behavioral abnormalities in *Ngl3*^−/−(Hyb)^ mice. We did not test whether Akt inhibition could correct the abnormal behaviors because the Akt inhibitor IV, although known to be membrane permeable, has been used only for in vitro studies [38–40], lacking information on blood-brain barrier (BBB) penetrance and pharmacokinetic properties.

**Fig 10:**
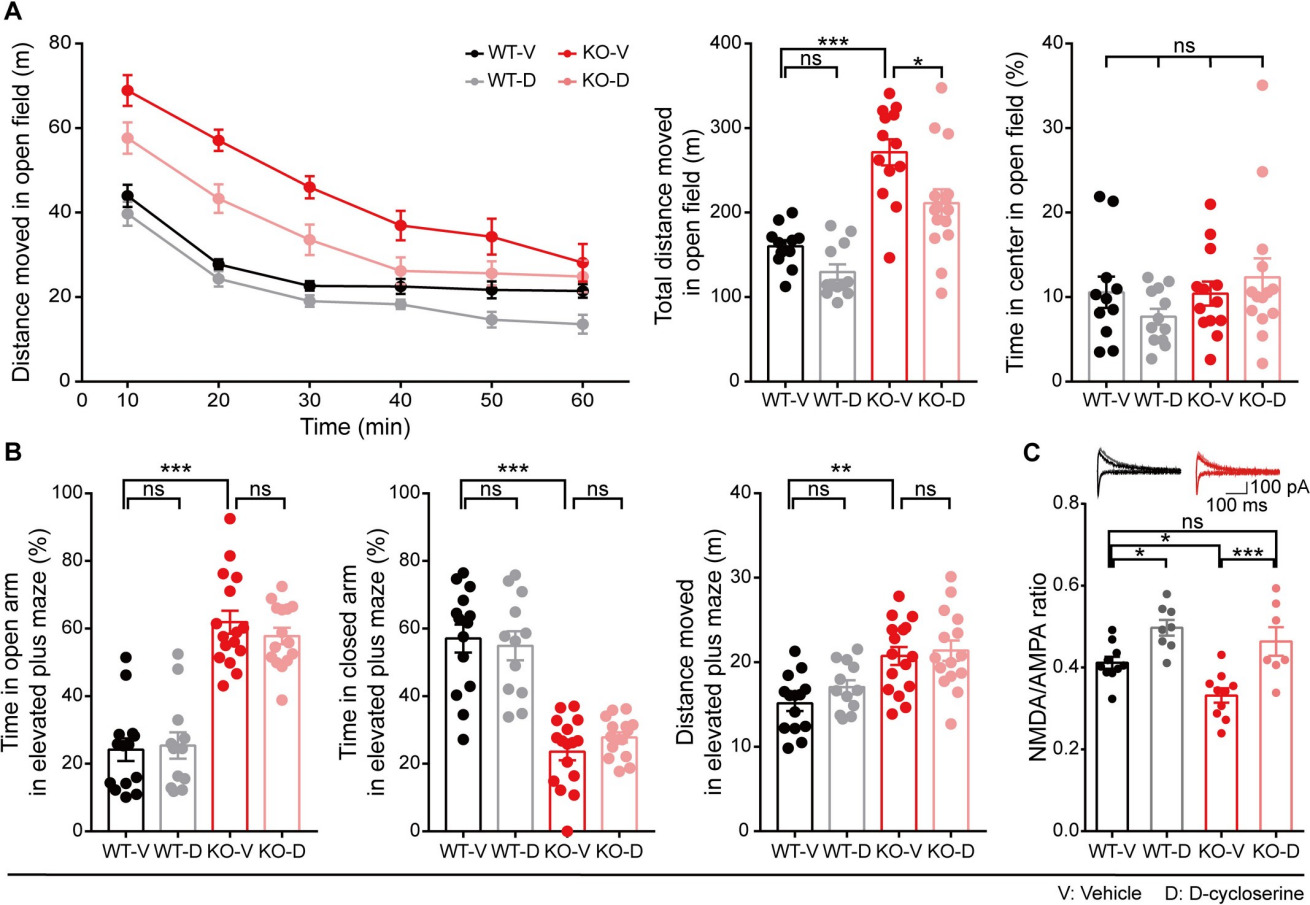
NMDAR activation rescues hyperactivity and NMDAR currents, but not anxiolytic-like behavior, in *Ngl3*^−/−(Hyb)^ mice. (A) DCS (20 mg/kg, intraperitoneal) or vehicle (saline), administered into the WT and *Ngl3*^−/−^ mice 30 minutes before the test, rapidly rescues the hyperactivity of *Ngl3*^−/−^ mice (2–3 months) without an effect on WT mice in the open-field test, as shown by the distance moved. *n* = 11 mice for WT-saline (V), 12 for WT-DCS (D), 13 for KO-V, and 15 for KO-D; *P < 0.05, ***P < 0.001, ns, not significant, two-way ANOVA with Bonferroni test. (B) DCS does not affect the anxiolytic-like behavior of *Ngl3*^−/−^ mice (2–3 months) in the elevated plus maze test, as shown by time in open/closed arms. Note that the hyperactivity of *Ngl3*^−/−^ mice on the elevated plus maze was not improved by DCS. *n* = 14 mice for WT-V, 12 for WT-D, 16 for KO-V, and 15 for KO-D; **P < 0.01, ***P < 0.001, ns, not significant, two-way ANOVA with Bonferroni test. (C) DCS rescues NMDAR function at SC-CA1 synapses of *Ngl3*^−/−^ mice (P16–20), as shown by the NMDA/AMPA ratio. *n* = 10 slices from seven mice for WT-V; 8, 6 for WT-D; 10, 9 for KO-V; and 7, 5 for KO-D; *P < 0.05, ***P < 0.001, ns, not significant, two-way ANOVA with Bonferroni test. Primary data can be found in S3 Data. AMPA, α-amino-3-hydroxy-5-methyl-4-isoxazolepropionic acid; DCS, D-cycloserine; KO-D, knockout, drug; KO-V, knockout, vehicle; NMDA, N-methyl-D-aspartate; NMDAR, NMDA receptor; ns, not significant; SC-CA1, Schaffer collateral-CA1 pyramidal; WT, wild-type; WT-D, wild-type, drug; WT-V, wild-type, vehicle.

Treatment of *Ngl3*^−/−(Hyb)^ mice with DCS (20 mg/kg, intraperitoneal) 30 minutes before the open-field test reduced hyperactivity in these animals (**Fig 10A**). In contrast, DCS did not affect the locomotor activity of WT mice. The effect of DCS on time spent in the center region (center time) could not be assessed because the center time after drug injection became comparable in vehicle-treated *Ngl3*^−/−(Hyb)^ and WT mice, suggesting sensitivity to handling or injection procedures. In another pharmacological rescue experiment, CDPPB (10 mg/kg, intraperitoneal), a positive allosteric modulator of metabotropic glutamate receptor 5 (mGluR5) that can potentiate NMDARs [55], similarly rescued the hyperactivity in *Ngl3*^−/−(Hyb)^ mice without affecting the center time (**S10 Fig**).

In contrast to the positive effect of DCS on hyperactivity in an open-field context, DCS did not improve the anxiolytic-like behaviors of *Ngl3*^−/−(Hyb)^ mice in the elevated plus maze test, as shown by the time spent in open and closed arms (**Fig 10B**). DCS also had no effect on the total distance moved in the elevated plus maze. Taken together, these results suggest that DCS rapidly rescues open-field hyperactivity in *Ngl3*^−/−(Hyb)^ mice. We did not test the effect of DCS on learning and memory impairments of *Ngl3*^−/−(Hyb)^ mice in the Morris water maze test because this test would have required repeated drug treatments during a large number of sessions over multiple days.

Finally, we tested whether DCS, which rescued hyperactivity in *Ngl3*^−/−(Hyb)^ mice, also rescues NMDAR function in these mice. DCS rapidly restored NMDAR function at SC-CA1 *Ngl3*^−/−(Hyb)^ synapses, as measured by the NMDA/AMPA ratio (**Fig 10C**), although it did not normalize LTP or LTD in the *Ngl3*^−/−(Hyb)^ hippocampus, as mentioned above (**Fig 5B and 5C**). Notably, DCS also increased NMDAR function at WT synapses, albeit to a lesser extent, although it had only minimal effects on the behavior of WT mice. These results collectively suggest that elevation of NMDAR-mediated currents, but not the correction of synaptic plasticity, rescues hyperactivity but not anxiolytic-like behavior in *Ngl3*^−/−(Hyb)^ mice.

## Discussion

Our data indicate that *Ngl3* KO in mice in the context of a pure genetic background has significant impacts on neurodevelopmental processes, affecting birth rate, postnatal growth and survival, and brain development (**Fig 1**). How might *Ngl3* KO exert such strong neurodevelopmental effects? NGL-3 is mainly expressed in the brain and directly interacts with important synaptic proteins, such as PSD-95 family proteins and presynaptic LAR-RPTPs [20–22]. However, mice lacking PSD-95 do not display severe neurodevelopmental deficits [56]. Instead, they show impaired spatial learning associated with abnormal synaptic plasticity that is qualitatively different from those observed in *Ngl3*^−/−(B6)^ mice; for instance, PSD-95–deficient mice show normal NMDAR-mediated synaptic transmission but enhanced LTP at all tested stimulation frequencies, including 1-Hz stimulation, which usually induces LTD [56]. Therefore, PSD-95 is less likely to be involved in the developmental phenotypes observed in *Ngl3*^−/−(B6)^ mice.

LAR-RPTPs are known to regulate not only synapse development but also early neurodevelopmental processes such as axon outgrowth and guidance [1, 4, 57]. In addition, mice lacking LAR-RPTPs such as PTPσ and PTPδ display severe developmental phenotypes, including peri- and postnatal semilethality and postnatal growth [58–60]. Moreover, our data indicate that synaptic levels of two LAR-RPTP family proteins (PTPδ and PTPσ) are decreased in *Ngl3*^−/−(B6)^ mice (**S2 Fig**). Therefore, *Ngl3* KO may dysregulate early developmental processes through the loss of *trans*-synaptic or interneuronal interactions of NGL-3 with LAR-RPTPs. In line with this possibility, NGL-3 proteins are detected at substantial levels at embryonic day 18 and P1 (earlier stages were not tested) in addition to relatively late postnatal stages in rats [22].

It has been shown that, in addition to interacting with NGL-3, LAR-RPTPs interact with other postsynaptic adhesion molecules, including TrkC, IL1RAPL1, IL1RAcP, Slitrks, and SALM3/5 [19, 21–30]. However, mice lacking some of these molecules, such as TrkC [61], IL1RAPL1 [62, 63], Slitrk1/3/5 [27, 64, 65], and SALM3 [29], do not show severe neurodevelopmental phenotypes involving altered birth rates, growth, or survival, although they do show relatively minor, but important, phenotypes such as deficits in synapse development, sensory function, and specific behaviors. These results suggest that, among many LAR-RPTP–interacting postsynaptic adhesion molecules, NGL-3 plays a more important role in the regulation of neurodevelopmental processes.

Then, why is NGL-3 more important for neurodevelopment processes among known LAR-RPTP–interacting postsynaptic adhesion molecules? Notably, our data associate *Ngl3* KO with enhanced Akt/GSK3β phosphorylation in the brain (**Fig 4**). Akt is a fundamental regulator of cellular development and function, including cell cycle regulation [66–68]. In the central nervous system, Akt has been extensively associated with brain development and neurodevelopmental disorders [69–71]. In addition, many substrates of Akt, including GSK3β, are implicated in brain development and disorders [72, 73]. These results suggest that *Ngl3* KO may impair brain development through altered Akt/GSK3β signaling.

Unexpectedly, and in contrast to in vitro results supporting the idea that NGL-3 promotes excitatory synapse development, *Ngl3* KO had minimal impacts on excitatory synapse development and AMPAR-mediated basal transmission (**Fig 2**). It is possible that the impacts of *Ngl3* KO may be dampened by other LAR-RPTP–interacting postsynaptic adhesion molecules. Notably, mice with TrkC knockdown [23] and those with IL1RAPL1 [62, 63] or SALM3 [29] KO show particularly strong reductions in excitatory synapses and dendritic spines, suggesting that these molecules have stronger influences on LAR-RPTP–dependent excitatory synapse development.

Our data indicate that *Ngl3* KO leads to moderate reductions in NMDAR-dependent synaptic transmission and LTP (**Fig 3**), reminiscent of other synaptic adhesion molecules that affect NMDARs, including neuroligin-1 [74], neuroligin-3 [75], leucine rich repeat transmembrane protein 2 (LRRTM2) [76], erythropoietin producing human hepatocellular receptor Bs or ephrin receptor Bs (EphBs) [77], and fibronectin leucine rich transmembrane protein 3 (FLRT3) [78]. A quantitative analysis indicated that LTP was decreased by approximately 32% and 40% in *Ngl3*^−/−(Hyb)^ and *Ngl3*^−/−(B6)^ mice, respectively, similar to the extent of the reduction in NMDAR-mediated transmission (approximately 28% and 18%, respectively). However, NMDAR activation by DCS does not rescue the suppressed LTP in *Ngl3*^−/−(Hyb)^ mice (**Fig 5B**), suggesting that reduced NMDAR currents do not suppress LTP. It is possible that some signaling pathways in the downstream of NMDAR activation that regulate synaptic delivery of AMPARs [79, 80] might have been changed. In addition, the mechanisms underlying the reduced NMDAR currents in *Ngl3*^−/−^ mice remain unclear. Our data suggest that the reduced NMDAR currents are unlikely to involve altered surface trafficking of NMDARs (**Fig 4C**) or changes in the subunit composition of NMDARs, based on the normal decay kinetics (**Fig 3A and 3B**). It is possible, again, the strongly altered Akt/GSK3β signaling and related signaling molecules might alter the phosphorylation and function of NMDARs [32] in *Ngl3*^−/−(Hyb)^ mice.

Intriguingly, LTD is almost completely abolished in *Ngl3*^−/−(Hyb)^ mice. This does not seem to involve reduced NMDAR currents because DCS does not rescue LTD (**Fig 5C**). Importantly, activities of Akt and GSK3β, signaling molecules known to regulate LTD [35, 36], are changed towards the direction that suppresses LTD (increased Akt and decreased GSK3β activities) (**Fig 4D**). In addition, Akt inhibition rapidly and strongly normalizes LTD in *Ngl3*^−/−(Hyb)^ slices (**Fig 5A**). Mechanisms underlying the strong changes in Akt/GSK3β signaling induced by *Ngl3* KO remain unclear. However, the C-terminal tail of NGL-3 directly interacts with PSD-95 family proteins [22] known to coordinate various synaptic signaling pathways [81, 82]. In addition, because LTD is known to refine neural circuits by redistributing synaptic proteins to more active synapses, it is conceivable that the near-complete elimination of LTD in *Ngl3*^−/−^ mice, which manifests in both genetic backgrounds, may contribute to the observed neurodevelopmental deficits and behavioral abnormalities.

*Ngl3* KO appears to be abnormally enhancing brain excitation in *Ngl3*^−/−(Hyb)^ mice, as evidenced by increases in sEPSC frequency (but not mEPSC frequency) and intrinsic neuronal excitability in the hippocampus, and increased susceptibility to induced seizures (**Fig 9**). This increase in the excitatory drive may represent a compensatory mechanism within the hippocampus to counteract the reduction in NMDAR function. Alternatively, it could be the consequences of *Ngl3* KO in brain regions other than the hippocampus. In support of this latter possibility, NGL-3 mRNA is widely expressed in various brain regions, including those enriched for GABAergic neurons, such as the striatum [17, 21]. In addition, our fluorescence in situ hybridization directly indicates that *Ngl3* mRNAs are detected in both excitatory and inhibitory neurons (**S8 Fig**). Therefore, *Ngl3* KO in inhibitory neurons might suppress the output functions of these neurons, resulting in the excitation of target excitatory neurons and the overall excitatory drive in the brain, although this does not seem to be the case in the hippocampus (normal mIPSCs and sIPSCs). However, we could observe altered alpha and theta oscillations in the temporal cortex of *Ngl3*^−/−(Hyb)^ mice, a result that may reflect disrupted interplay of excitatory and inhibitory neurons.

Behaviorally, *Ngl3*^−/−(Hyb)^ mice display abnormal hyperactivity, anxiolytic-like behaviors, and impaired spatial and motor learning and memory; however, recognition learning and memory were unchanged (**Figs 6–8** and **S5 Fig**). These results suggest that, although NGL-3 is widely expressed in various brain regions, *Ngl3* KO leads to specific behavioral deficits. We also found that pharmacological enhancement of NMDAR currents by acute DCS treatment normalizes hyperactivity, but not anxiolytic-like behavior, in *Ngl3*^−/−(Hyb)^ mice (**Fig 10**). Although DCS did not rescue LTP or LTD (**Fig 5B and 5C**), enhanced NMDAR currents would increase the excitability of postsynaptic dendrites and neurons, increasing their output functions and disrupting related neural circuits [52–54].

Although NGL-3 by itself has not been directly implicated in any particular brain disorders, its presynaptic LAR-RPTP binding partners have been extensively linked to various psychiatric disorders [1, 4]. For example, PTPδ has been associated with restless leg syndrome [42–44], ADHD [45], ASD [46], and bipolar disorder [47]. Given that deletion of *trans*-synaptic partners (e.g., NGL-2 and its presynaptic ligand netrin-G2) in mice often results in phenotypic similarities [83–86], the abnormalities at the synapse, systems, and behavioral levels observed in *Ngl3*^−/−^ mice may help us understand how LAR-RPTPs are associated with specific psychiatric disorders.

Associating all the phenotypes observed in *Ngl3*^−/−(Hyb)^ and *Ngl3*^−/−(B6)^ mice is a great challenge, but the following questions could be raised. How does the change in the genetic background dramatically improve brain development and mouse survival? How do the changes in synaptic signaling and synaptic plasticity, in particular in LTD, impair multiple behaviors in the mutant mice? The beneficial effects of a hybrid background on brain development and survival may be attributable to the suppression of the homozygosity of certain genetic traits under a single genetic background that causes certain harmful effects. Indeed, it has been shown that mice in hybrid genetic backgrounds show higher viability and better performance in behavioral tests such as complex learning and memory tasks [87–89]. Identification of specific chromosomal loci or genes using genetic methods such as quantitative trait loci (QTL) analyses, genome wide association studies (GWAS), and exome/genome sequencing would eventually lead to the identification of related mechanisms, although they would have to be pursued in future studies. The impaired LTD during the critical period of activity-dependent excitatory synapse and circuit refinement during brain development might suppress the sharpening process of certain synapses and circuits required for specific behaviors. In addition, the altered Akt/GSK3β signaling in *Ngl3*^−/−^ mice, known to affect numerous synaptic and non-synaptic target proteins and be associated with various brain disorders, including schizophrenia, Alzheimer disease, and bipolar disorder [72, 90–94], may alter the synaptic, neuronal, and circuit functions.

It remains unknown whether mutations in the human *LRRC4B* gene encoding NGL-3 are associated with brain disorders. However, presynaptic binding partners of NGL-3 such as PTPδ and PTPσ have been implicated in multiple brain disorders, including restless leg syndrome, attention deficit/hyperactivity disorder, autism spectrum disorder, bipolar disorder, Alzheimer disease, obsessive compulsive disorder, addiction, and mood liability [1, 4, 95]. In addition, NGL-3 has been reported as a novel and LTD-dependent substrate of matrix metalloproteinases and the presenilin/γ-secretase complex [96], known to be associated with diverse brain disorders including Alzheimer disease and multiple sclerosis [97–100]. It is therefore possible that NGL-3 might contribute to some of the pathophysiological mechanisms underlying these brain disorders.

In conclusion, our results suggest that NGL-3 regulates neurodevelopment, Akt/GSK3β signaling, LTD, brain excitation, and specific behaviors.

## Materials and methods

### Ethics statement

We used isoflurane anesthetization for slice preparation, and we anesthetized mice with Avertin (tribromoethanol, 20 mg/mL) for EEG.

Mice were bred and maintained according to the Requirements of Animal Research at Korea Advanced Institute of Science and Technology (KAIST), and all procedures were approved by the Committee of Animal Research at KAIST (KA2016-27).

### Generation and characterization of *Ngl3*^−/−^ mice

Exon 2 of the *Ngl3* mice was replaced by a cassette containing the β-geo-puromycin gene and a polyadenyl action signal by homologous recombination, which were generated by Lexicon (Stamford, CT) and obtained from Taconic (Rensselaer, NY) (TF2916; F1 generation). We crossbred these mice with C57BL/6J and 129/Sv mice in parallel at least for five generations. *Ngl3*^−/−(Hyb)^ mice were produced by crossbreeding just before experiments. Mice were housed in a standard cage environment under 12-hour light and dark cycles.

### Animal behavioral test

Mice used in all behavioral tests were 2–5 months old. All assays used littermates or age-matched animals. All behavioral results were analyzed in a blind manner.

### Pharmacological rescue

For the rescue of LTD by Akt inhibition, the Akt inhibitor IV (Cayman chemical, 10 μM) was added to ACSF, and hippocampal slices were exposed to ACSF with Akt inhibitor IV at least 30 minutes before recording. For vehicle treatment, DMSO was added to ACSF. DCS (Sigma, St. Louis, MO) was dissolved in saline to a final concentration of 5 g/L. WT and *Ngl3*^−/−(Hyb)^ mice received an intraperitoneal injection of DCS (20 mg/kg), or the same volume of saline, 30 minutes before behavioral tests. For rescue experiments of the NMDA/AMPA ratio, LTP, LTD, and NMDAR mEPSC in slice preparations, DCS (20 μM) was added to ACSF. Hippocampal slices were exposed to ACSF with DCS at least 30 minutes before recording. For vehicle treatment, 100 μL of saline was added to ACSF. CDPPB (Ascent Scientific, Cambridge, United Kingdom) was dissolved in DMSO and PEG400 (DMSO:PEG400 = 1:9) to a final concentration of 6 g/L. WT and *Ngl3*^−/−(Hyb)^ mice received an intraperitoneal injection of CDPPB (10 mg/kg) or the same volume of DMSO, PEG mixture, 30 minutes before behavioral tests.

### EEG

Mice were anesthetized with Avertin (tribromoethanol, 20 mg/mL) and placed in a stereotaxic device. Four epidural electrodes for EEG recordings were implanted with connectors (Omnetics, Minneapolis, MN) in the frontal (2.8 mm anterior and 1.6 mm lateral to bregma) and temporal cortexes (2.4 mm posterior and 1.6 mm lateral from bregma). A grounding electrode was implanted in the occipital region of the skull. After 7 days of recovery, EEG signals were recorded for 60 minutes, during which mice were allowed to freely explore their home cages.

### Statistics

Gender, number of mice used, and details of the statistical results are described in **S1 Data**. The data with nonparametric distribution were analyzed by Mann-Whitney test, and those with parametric distribution were analyzed by Student *t* test. If the data are parametric but have a significant difference in variance in the F test, Welch correction was used. GraphPad Prism 7 was used for statistical analysis.

Additional methods can be found in **S2 Data**.

The numerical data used in all figures are included in **S3 Data**.

## Supporting information

S1 Data Statistical results. (XLSX)

S2 Data Supplementary methods. (DOCX)

S3 Data Numerical raw data. Excel spreadsheet containing, in separate sheets, the underlying numerical data and statistical analysis for figure panels 1A, 1B, 1B, 2A, 2B, 2C, 2D, 2E, 2F, 3A, 3B, 3C, 3D, 3E, 3F, 4A, 4B, 4C, 4D, 5A, 5B, 5C, 6A, 6B, 6C, 6D, 6E, 6F, 7A, 7B, 7C, 7D, 8A, 8B, 8C, 8D, 8E, 8F, 9A, 9B, 9C, 9D, 9E, 10A, 10B, 10C, S2A, S2B, S4A, S4B, S4C, S5A, S5B, S5C, S5D, S5E, S5F, S5G, S6A, S6B, S6C, S6D, S6E, S6F, S7A, S7B, S7C, S7D, S7E, S7F, S7G, S7H, S7I, S9A, S9B, S9C, S9D, and S10A.
(XLSX)

S1 Fig Generation and characterization of *Ngl3*^−/−^ mice in a C57BL/6J background. (A) Schematic diagram of the *Ngl3* gene KO strategy. (B) PCR genotyping of *Ngl3*^−/−^ mice. (C) Lack of detectable NGL-3 protein in whole-brain lysates of *Ngl3*^−/−(B6)^ mice (8 weeks). (D) A schematic showing production of *Ngl3*^−/−^ mice in two different genetic backgrounds: a pure C57BL/6J background (*Ngl3*^−/−(B6)^) and a hybrid 129/Sv + C57B/6J background (*Ngl3*^−/−(Hyb)^). HT, heterozygous; KO, knockout; NGL-3, Netrin-G ligand-3.
(TIF)

S2 Fig Normal levels of NGL-3 relatives but suppressed levels of PTPδ in the *Ngl3*^−/−(Hyb)^ brain. (A and B) Synaptic levels of NGL-3 relatives (NGL-1 and NGL-2) and NGL-3–binding presynaptic adhesion molecules (PTPδ and PTPσ) were also tested by immunoblot analysis of crude synaptosomes of the *Ngl3*^−/−^ brain (3 and 10 weeks). Note that levels of PTPδ are significantly reduced at postnatal weeks 3 and 10. *n* = 4 mice for WT and KO, *P < 0.05, **P < 0.01, Student *t* test. Primary data can be found in S3 Data. KO, knockout; NGL, Netrin-G ligand; PTPδ, protein tyrosine phosphatase δ; PTPσ, protein tyrosine phosphatase σ; WT, wild-type.
(TIF)

S3 Fig Distribution patterns of NGL-3 proteins revealed by X-gal staining and normal excitatory and inhibitory synaptic signals in the *Ngl3*^−/−(Hyb)^ hippocampus. (A) Distribution patterns of NGL-3 proteins, revealed by X-gal staining of *Ngl3*^+/–(Hyb)^ coronal brain slices (8–10 weeks). Scale bar, 1 mm. (B and C) Staining of VGluT1 (excitatory presynaptic marker) and VGAT (inhibitory presynaptic marker) in the hippocampus in *Ngl3*^−/−(Hyb)^ mice (10 weeks). NGL-3, Netrin-G ligand-3; VGAT, vesicular GABA transporter; VGluT1, vesicular glutamate transporter 1.
(TIF)

S4 Fig *Ngl3*^−/−(Hyb)^ mice show normal inhibitory spontaneous synaptic transmission and mGluR LTD, but not in NMDAR mEPSC. (A) Normal mIPSCs in hippocampal CA1 neurons of *Ngl3*^−/−(Hyb)^ mice (P21–23). *n* = 15 cells from three mice for WT and KO; ns, not significant, Student *t* test. (B) Suppressed frequency and amplitude of NMDAR mEPSCs in hippocampal CA1 neurons of *Ngl3*^−/−(Hyb)^ mice (P19–20). *n* = 14 cells from three mice for WT and 13, 3 for KO; *P < 0.05, Student *t* test. (C) Normal mGluR-LTD induced by DHPG treatment (50 μM) at hippocampal SC-CA1 synapses of *Ngl3*^−/−(Hyb)^ mice (P16–20). *n* = 8, 4 for WT and 8, 3 for KO; ns, not significant, Student *t* test. Primary data can be found in S3 Data. CA1, Cornu Ammonis 1; DHPG, (RS)-3,5-dihydroxyphenylglycine; KO, knockout; mEPSC, miniature excitatory postsynaptic current; mGluR, metabotropic glutamate receptor; LTD, long-term depression; mIPSC, miniature inhibitory postsynaptic current; NMDAR, NMDA receptor; ns, not significant; P, postnatal day; SC-CA1, Schaffer collateral-CA1 pyramidal; WT, wild-type.
(TIF)

S5 Fig *Ngl3*^−/−(B6)^ and *Ngl3*^−/−(B6)^ mice show normal social interaction and novel-object recognition. (A) Normal social interaction and social novelty recognition in *Ngl3*^−/−(Hyb)^ mice (2–4 months) in the three-chamber social interaction test, as shown by time spent sniffing. *n* = 7 mice for WT and 6 for KO. ***P < 0.001, ns, not significant, one-way ANOVA with Tukey multiple comparison test. (B) Normal self-grooming and marble burying in *Ngl3*^−/−(Hyb)^ mice (2–4 months). *n* = 11 mice for WT and 12 for KO for self-grooming, *n* = 14 mice for WT and 11 for KO for marble burying test; ns, not significant, Student *t* test. (C) Normal social interaction and social novelty recognition of *Ngl3*^−/−(B6)^ mice (2–4 months) in the three-chamber social interaction test, as shown by time spent sniffing. *n* = 7 mice for WT and 6 for KO; *P < 0.05, **P < 0.01, ***P < 0.001, one-way ANOVA with Tukey test. (D) Normal object recognition memory in *Ngl3*^−/−(Hyb)^ mice (2–4 months) in the novel-object recognition test. *n* = 15 mice for WT and 11 for KO; ns, not significant, Student *t* test. (E) Normal fear memory of *Ngl3*^−/−(Hyb)^ mice (2–4 months) in the contextual fear conditioning test. *n* = 13 mice for WT and 10 for KO; ns, not significant, Student *t* test. (F) Normal object recognition of *Ngl3*^−/−(B6)^ mice (2–4 months) in the novel-object recognition test. *n* = 15 mice for WT and 11 for KO; ns, not significant, Student *t* test. (G) Impaired motor learning of *Ngl3*^−/−(Hyb)^ mice (2–4 months) in the rotarod test. *n* = 11 mice for WT and 12 for KO; ***P < 0.001, two-way ANOVA with Bonferroni test. Primary data can be found in S3 Data. KO, knockout; ns, not significant; WT, wild-type.
(TIF)

S6 Fig Heterozygosity of *Ngl3* in mice does not affect synaptic transmission or plasticity in the hippocampus. (A and B) Normal mEPSCs and mIPSCs in *Ngl3*^+/–(B6)^ hippocampal CA1 neurons (P17–23 for mEPSCs and P18–22 for mIPSCs). *n* = 16 cells from five mice for WT and 13, 5 for KO (mEPSC); 12, 4 for WT and 15, 5 for KO (mIPSC); ns, not significant, Student *t* test. (C and D) Normal input-output relationship and paired-pulse ratio at *Ngl3*^+/–(B6)^ hippocampal SC-CA1 synapses (P27–29), as shown by fEPSP slopes plotted against fiber volley amplitudes and paired-pulse ratios plotted against inter-pulse intervals. *n* = 10 cells from three mice for WT and KO for both input-output and paired-pulse ratio, two-way ANOVA with Bonferroni test. (E) Normal NMDAR function at *Ngl3*^+/–(B6)^ hippocampal SC-CA1 synapses (P17–23), as shown by the NMDA/AMPA ratio. *n* = 8 cells from five mice for WT and 8, 7 for KO; ns, not significant, Student *t* test. (F) Normal LTP induced by HFS (100 Hz, 1 second) at *Ngl3*^+/–(B6)^ hippocampal SC-CA1 synapses (P23–33). *n* = 8 slices from six mice for WT and 10, 7 for KO; ns, not significant, Student *t* test. Primary data can be found in S3 Data. AMPA, α-amino-3-hydroxy-5-methyl-4-isoxazolepropionic acid; CA1, Cornu Ammonis 1; fEPSP, field excitatory postsynaptic potential; KO, knockout; LTP, long-term potentiation; mEPSC, miniature excitatory postsynaptic current; mIPSC, miniature inhibitory postsynaptic current; NMDA, N-methyl-D-aspartate; NMDAR, NMDA receptor; P, postnatal day; SC-CA1, Schaffer collateral-CA1 pyramidal; WT, wild-type.
(TIF)

S7 Fig Heterozygous *Ngl3*^+/–(B6)^ mice show moderate hypoactivity and anxiolytic-like behavior. (A) Moderate hypoactivity of *Ngl3*^+/–(B6)^ mice (2–4 months) in the open-field test. Mean ± SEM. *n* = 20 mice for WT and heterozygote (HT); *P < 0.05, two-way ANOVA with Bonferroni test and Student *t* test. (B) Normal locomotor activity of *Ngl3*^+/–(B6)^ mice (2–4 months) in the Laboras test, in which mouse movements are continuously monitored for 48 hours. *n* = 15 mice for WT and HT; ns, not significant, two-way ANOVA with Bonferroni test and Student *t* test. (C) Normal anxiety-like behavior of *Ngl3*^+/–(B6)^ mice (2–4 months) in the elevated plus maze test, as shown by time spent in and entries into open/closed arms. *n* = 9 mice for WT and 8 for HT; ns, not significant, Student *t* test. (D) Moderate anxiolytic-like behavior of *Ngl3*^+/–(B6)^ mice (2–4 months) in the light-dark test, as shown by transition number and chamber time. *n* = 18 mice for WT and 17 for HT; *P < 0.05, ns, not significant, Student *t* test. (E) Normal object memory of *Ngl3*^+/–(B6)^ mice (2–4 months) in the novel-object recognition test. *n* = 20 mice for WT and 19 for HT; ns, not significant, Student *t* test. (F) Normal motor learning of *Ngl3*^+/–(B6)^ mice (2–4 months) in the rotarod test. *n* = 10 mice for WT and HT; ns, not significant, repeated measure of ANOVA. (G) Normal social interaction and social novelty recognition of *Ngl3*^+/–(B6)^ mice (2–4 months) in the three-chamber social interaction test, as shown by time spent in sniffing. *n* = 12 mice for WT and 10 for HT; ***P < 0.001, one-way ANOVA with Tukey test. (H) Normal spatial memory of *Ngl3*^+/–(B6)^ mice (2–4 months) in the learning, probe, and reversal phases of the Morris water maze test. Quadrant occupancy during the probe phase is also indicated. *n* = 10 mice for WT and 9 for HT; **P < 0.01, ns, not significant, two-way ANOVA with Bonferroni test and Student *t* test. (I) Normal fear memory of *Ngl3*^+/–(B6)^ mice (2–4 months) in the contextual fear conditioning test. *n* = 12 mice for WT and KO; ns, not significant, Student *t* test. Primary data can be found in S3 Data. HT, heterozygote; ns, not significant; WT, wild-type.
(TIF)

S8 Fig Localization of *Ngl3/Lrrc4b* mRNAs in both excitatory and inhibitory neurons. (A and B) Localization of *Ngl3/Lrrc4b* mRNAs in both excitatory and inhibitory neurons in the cortex and hippocampus of WT mice (P56), as determined by fluorescence in situ hybridization and shown by the colocalization of Ngl3/Lrrc4b mRNAs and Vglut1/2 (excitatory neuronal marker) or Gad1/2 (inhibitory neuronal marker). Arrowheads indicate examples of neurons that express both Ngl3/Lrrc4b and Vglut1/2 or Gad1/2 mRNAs. Scale bar, 0.2 mm (left) and 20 μm (right). Gad1/2, glutamate decarboxylase 1/2; Ngl3/Lrrc4b, Netrin-G ligand-3/Leucine-rich repeat-containing protein 4B; P, postnatal day; Vglut1/2, vesicular glutamate transporter 1/2; WT, wild-type.
(TIF)

S9 Fig Brain oscillations in *Ngl3*^−/−(Hyb)^ mice. (A-D) Total power of brain oscillations (A) and brain oscillations in different frequency ranges, normalized to the total power (B–D). Note that none of the comparisons yielded significant differences except alpha and theta ranges in the temporal lobe (see main figure panels). *n* = 6 mice for WT and KO; ns, not significant, Student *t* test. Primary data can be found in S3 Data. KO, knockout; ns, not significant; WT, wild-type.
(TIF)

S10 Fig CDPPB rescues hyperactivity in *Ngl3*^−/−(Hyb)^ mice. (A) CDPPB (10 mg/kg, intraperitoneal), administered 30 minutes before the test, rapidly rescues the hyperactivity of *Ngl3*^−/−^ mice (2–3 months) in the open-field test, as shown by the distance moved. *n* = 11 mice for WT-saline (V), 12 for WT-DCS (D), 17 for KO-V, and 18 for KO-D; *P < 0.05, ***P < 0.001, ns, not significant, two-way ANOVA with Bonferroni test. Primary data can be found in S3 Data. DCS, D-cycloserine; KO-D, knockout, drug; KO-V, knockout, vehicle; ns, not significant; V, vehicle; WT, wild-type.
(TIF)

## Abbreviations

AMPA: α-amino-3-hydroxy-5-methyl-4-isoxazolepropionic acid
AMPAR: AMPA receptor
ASD: autism spectrum disorder
BBB: blood-brain barrier
CA1: Cornu Ammonis 1
DCS: D-cycloserine
EphB: erythropoietin producing human hepatocellular receptor B
EPSC: excitatory postsynaptic current
fEPSP: field excitatory postsynaptic potential
FLRT3: fibronectin leucine rich transmembrane protein 3
β-geo: β-galactosidase + neomycin resistance
GluN1: Glutamate, NMDAR subunit 1
GluN2A: Glutamate, NMDAR subunit 2A
GluN2B: Glutamate, NMDAR subunit 2B
GSK3β: glycogen synthase kinase 3β
GWAS: genome wide association studies
HE: hematoxylin–eosin
HFS: high-frequency stimulation
IL1RAcP: interleukin 1 receptor accessory protein
IL1RAPL1: interleukin 1 receptor accessory protein like 1
KAIST: Korea Advanced Institute of Science and Technology
KO: knockout
LAR: leukocyte common antigen-related
LAR-RPTP: LAR family receptor tyrosine phosphatase
LFS: low-frequency stimulation
LRRTM2: leucine rich repeat transmembrane protein 2
LTD: long-term depression
LTP: long-term potentiation
MAP2: microtubule associated protein 2
mEPSC: miniature excitatory postsynaptic current
mGluR: metabotropic glutamate receptor
mGluR5: metabotropic glutamate receptor 5
mIPSC: miniature inhibitory postsynaptic current
mTOR: mammalian target of rapamycin
mTORC2: mTOR complex 2
NBQX: 2,3-Dioxo-6-nitro-1,2,3,4-tetrahydrobenzo(f)quinoxaline-7-sulfonamide
NGL-3: Netrin-G ligand-3
NGL/LRRC4: Netrin-G ligand
NMDA: N-methyl-D-aspartate
NMDAR: NMDA receptor
P: postnatal day
PDK1: 3-phosphoinositide-dependent protein kinase 1
PI3K: phosphatidyl inositol 3 kinase
PKC: protein kinase C
PSD-95: postsynaptic density-95
PTPδ: protein tyrosine phosphatase δ
PTPσ: protein tyrosine phosphatase σ
PTZ: pentylenetetrazol; QTL, quantitative trait loci
SALM: synaptic adhesion-like molecule
SC-CA1: Schaffer collateral-CA1 pyramidal
sEPSC: spontaneous EPSC
sIPSC: spontaneous IPSC
Slitrk1–5: Slit- and Trk-like proteins, VGAT, vesicular GABA transporter
VGlut1: vesicular glutamate transporter 1
WT: wild-type
