# Supplementary material for: NGL-3 in the regulation of brain development, Akt/GSK3b signaling, long-term depression, and locomotive and cognitive behaviors
Source: PLoS Biol. 2019 Jun 5;17(6):e2005326. doi: 10.1371/journal.pbio.2005326 (PMC6550391; doi:10.1371/journal.pbio.2005326)
Supplement: S2 Data — (DOCX) [file pbio.2005326.s002.docx]

**NGL-3 in the regulation of brain development, Akt/GSK3β signaling, long-term depression, and locomotive and cognitive behaviors**

Hyejin Lee,^1,#^ Wangyong Shin,^2,#^ Kyungdeok Kim,^2,#^ Suho Lee,^1^ Eun-Jae Lee,^3^ Jihye Kim,^1^ Hanseul Kweon,^2^ Eunee Lee,^1^ Haram Park,^2^ Muwon Kang,^2^ Esther Yang,^4^ Hyun Kim,^4^ and Eunjoon Kim^1,2,*^

^1^Center for Synaptic Brain Dysfunctions, Institute for Basic Science (IBS), Daejeon 305-701, Korea; ^2^Department of Biological Sciences, Korea Advanced Institute for Science and Technology (KAIST), Daejeon 305-701, Korea; ^3^Department of Neurology, Asan Medical Center University of Ulsan, College of Medicine, Seoul, South Korea; ^4^Department of Anatomy, College of Medicine, Korea University, Seoul 136-705, Korea; ^#^These authors contributed equally to the study; *Corresponding author.

**Supplementary methods**

## Electrophysiology

For hippocampal electrophysiological experiments, sagittal hippocampal slices (400 μm thickness for extracellular recordings and 300 μm for intracellular recordings) of the mutant mice (Ngl3^–/–(B6)^ mice, Ngl3^–/–(Hyb)^ mice, Ngl3^+/–(B6)^ mice) and their WT littermates were prepared using a vibratome (Leica VT1200) in ice-cold dissection buffer containing (in mM) 212 sucrose, 25 NaHCO_3_, 5 KCl, 1.25 NaH_2_PO_4_, 0.5 CaCl_2_, 3.5 MgSO_4_, 10 D-glucose, 1.25 L-ascorbic acid and 2 Na-pyruvate bubbled with 95% O_2_/5% CO_2_. The slices were recovered at 32 °C for 1 h in normal ACSF (in mM: 125 NaCl, 2.5 KCl, 1.25 NaH_2_PO_4_, 25 NaHCO_3_, 10 glucose, 2.5 CaCl_2_ and 1.3 MgCl_2_ oxygenated with 95% O_2_/5% CO_2_). For the recording, a single slice was moved to and maintained in submerged-type chamber at 28 °C, continuously perfused with ACSF (2 ml/min) saturated with 95% O_2_/5% CO_2_. Stimulation and recording pipettes were pulled from borosilicate glass capillaries (Harvard Apparatus) using a micropipette electrode puller (Narishege).

For extracellular recordings, mouse hippocampal slices at the age of postnatal day 16–33 were used. fEPSPs were recorded in the stratum radiatum of the hippocampal CA1 region using pipettes filled with ACSF (1 MΩ). fEPSP was amplified (Multiclamp 700B, Molecular Devices) and digitized (Digidata 1440A, 1550 Molecular Devices) for measurements. The Schaffer collateral pathway was stimulated every 20 s with pipettes filled with ACSF (0.3–0.5 MΩ).­­­­ The stimulation intensity was adjusted to yield a half-maximal response, and three successive responses were averaged and expressed relative to the normalized baseline. To induce LTP and LTD, high-frequency stimulation (100 Hz, 1 s) and low-frequency stimulation (1 Hz, 15 min) were applied after a stable baseline was acquired, respectively. To induce mGluR dependent LTD, DHPG (50 μM) was added to ACSF for 10 min after acquiring a stable baseline. Paired pulse ratio was measured across a range of inter-stimulus intervals of 25, 50, 75, 100, 200, and 300 ms.

Whole-cell patch-clamp recordings of hippocampal CA1 pyramidal neurons were made using a MultiClamp 700B amplifier (Molecular Devices) and Digidata 1440A, 1550 (Molecular Devices). During whole-cell patch-clamp recordings, series resistance was monitored each sweep by measuring the peak amplitude of the capacitance currents in response to short hyperpolarizing step pulse (5 mV, 40 ms); only cells with a change in < 20% were included in the analysis. For afferent stimulation of hippocampal pyramidal neurons, the Schaffer collateral pathway was selected. For NMDA/AMPA ratio experiments, mouse hippocampal slices (P19-P23) were used. The recording pipettes (2.5–3.5 MΩ) were filled with an internal solution containing the following (in mM): 100 CsMeSO_4_, 10 TEA-Cl, 8 NaCl, 10 HEPES, 5 QX-314-Cl, 2 Mg-ATP, 0.3 Na-GTP and 10 EGTA, with pH 7.25, 295 mOsm. CA1 pyramidal neurons were voltage clamped at -70 mV, and EPSCs were evoked at every 15 s. AMPAR-mediated EPSCs were recorded at -70 mV, and 20 consecutive responses were recorded after stable baseline. After recording AMPAR-mediated EPSCs, holding potential was changed to +40 mV to record NMDAR-mediated EPSCs. NMDA component was measured at 60 ms after the stimulation. The NMDA/AMPA ratio was determined by dividing the mean value of 20 NMDA components of EPSCs by the mean value of 20 AMPAR-mediated EPSC peak amplitudes.

Somatic whole-cell recording of mEPSCs were obtained in hippocampal CA1 pyramidal neurons at a holding potential of -70 mV. TTX (1 μM) and picrotoxin (100 μM) were added to ACSF to inhibit spontaneous action potential-mediated synaptic currents and IPSCs, respectively. For the recording of sEPSCs, only picrotoxin (100 mΜ) were added to ACSF. For NMDAR-mediated mEPSCs, CaCl_2_ and MgCl_2_ in ACSF were replaced with SrCl_2_ (4Mm), and NBQX (10 μM) were added to ACSF. For hippocampal CA1 pyramidal neuron mIPSCs, recording pipettes (2.5-3.5 MΩ) were filled with an internal solution containing the following (in mM): 120 CsCl, 10 TEA-Cl, 8 NaCl, 10 HEPES, 5 QX-314-Cl, 4 Mg-ATP, 0.3 Na-GTP and 10 EGTA, with pH 7.35, 280 mOsm. TTX (1 μM), NBQX (10 μM) and D-AP5 (50 μM) were added to ACSF to inhibit spontaneous action potential-mediated synaptic currents, AMPAR-mediated currents and NMDAR-mediated currents, respectively. For the recording of sIPSCs, NBQX (10 μM) and D-AP5 (50 μM) were added to ACSF. To measure excitability of hippocampal CA1 cell, recording pipettes (2.5 -3.5 MΩ) were filled with an internal solution containing the following (in mM): 137 K-Gluconate, 5 KCl, 10 HEPES, 0.2 EGTA, 10 Na-phosphocreatine, 4 Mg-ATP, and 0.5 Na-GTP, with pH 7.2, 280 mOsm. To inhibit postsynaptic responses, picrotoxin (100 μM), NBQX (10 μM) and D-AP5 (50 μM) were added. After rupturing the cell, the currents were clamped, and RMP was measured. Cells with RMP larger than -60 mV were not used. After stabilizing cell, RMP was adjusted by -65 mV. Current input was increased from 0 to 360 in increments of 30 pA per sweep. Each current was injected with a time interval of 15 seconds.

Data were acquired by Clampex 10.2 (Molecular Devices) and analyzed by Clampfit 10 (Molecular Devices). Drugs were purchased from Abcam (TTX), Tocris (NBQX, D-AP5) and Sigma (picrotoxin, DCS).

## Brain fractionation

Crude synaptosomes were prepared as described ^1^. Briefly, mouse brains (2 months old) were homogenized in ice-cold homogenization buffer (0.32 M sucrose, 10 mM HEPES, pH 7.4, 2 mM EDTA, protease inhibitors and phosphatase inhibitors). The homogenates were centrifuged at 1100 x g for 10 min. The resulting supernatant was centrifuged again at 12,000 x g for 15 min. The pellet was resuspended in homogenization buffer and centrifuged at 13,000 x g for 15 min (the resulting pellet is P2; crude synaptosomes). This sample was immunoblotted with various antibodies.

## Antibodies

Guinea pig antibodies against NGL-1 (#2041; guinea pig), NGL-2 (#2044; guinea pig), PTPδ (#2063; guinea pig), and PTPσ (#2135; guinea pig) were generated using synthetic peptides mimicking the last 30 aa of mouse NGL-1 and NGL-2 (VNTINSIHSSVHEPLLIRMNSKDNVQETQI and GNSLHPTVTTISEPYIIQTHTKDKVQETQI), aa 362-399 of mouse PTPδ (**IIQHKPKNSEEPYKEIDGIATTRYSVAGLSPYSDYEFR**) and aa 30-43 of mouse PTPσ (**EEPPRFIREPKDQIGVSGGVASFVC**) as immunogens. The following antibodies have been described: EGFP (#1168 ^2^ and #1997 ^3^), GluA1 (#1193), GluA2 (#1195) and NGL-3 (#2020 and #2021) ^4^. The following antibodies were purchased commercially: GluN1, PKC (BD Transduction Laboratories), phosphor-GluN1 (Ser896), GluN2A (Millipore), GluN2B (NeuroMab), phospho-GluN2B (Ser1284), phospho-GluN2B (Tyr1472), PI3K, phosphor-PI3K (Tyr458), phosphor-Akt (Tyr308), Akt, phosphor-PKC (Ser660), GSK3b, phosphor-GSK3b (Ser9) (Cell Signaling), phospho-GluN2B (Tyr1336), phospho-GluN2B (Ser1480) (Abcam), α-tubulin, Flag, HA, Myc (Sigma). For immunohistochemistry, VGluT1 (Sigma) and VGAT (Sigma) antibodies were used.

**Tissue biotinylation**

For tissue biotinylation, following paper previously described the method very well ^5^. For details, six slices per each mouse were prepared as same as electrophysiology, followed by dissecting only hippocampal formations. After sections are recovered for 40 min in 32°C, they are incubated for 1 hr with 2mg / ml EZ-link sulfo-NHS-LC-Biotin (PIERCE) in aCSF after clearing all existing aCSF. Biotin solution is washed 3 times for 10 min each. The remaining biotin is quenched with aCSF containing 100mM glycine through incubating for 20 min two times. After washing quenching solution by washed with aCSF three times of 10 min each, slices were centrifuged and aCSF was removed. 400 ul of lysis buffer (1% Triton-X 100, 150mM NaCl, 50mM Tris-pH 7.4, 1mM EDTA with protease inhibitor cocktails) was added, and they were dissolved by harsh pipetting and followed 30 min shaking incubation in 4°C. The amount of protein is normalized by BCA and among them, 270 ug of protein is used for the avidin-IP. Together with lysate, 100ul of NeutrAvidin Agarose Resin (Thermo Scientific) was incubated in 4°C overnight with shaking. After washing the bead for 3 times, 40ul of 2xSDS sample buffer was added and boiled in 65'C for 30 min. 27ug of protein is set for input (10%). Input was finally prepared by adding the same amount of 2xSDS sample buffer and same courses of boiling. 50% of immunoprecipitated (20 ul) and 45% input proteins (depends on sample amount) were used for WB analysis. Surface GluN1, GluA1 were normalized total GluN1, GluA1 intensity by normalized by tubulin.

## Open field test

Mice were placed in an open field box (40 x 40 x 40 cm) and recorded with a video camera for 60 min. The center zone line was 10 cm apart from the edge. The testing room was illuminated at ~50 lux or 0 lux. Mice movements were analyzed using EthoVision XT 10 program (Noldus).

## Automated 48-hr movement analysis

For a long-term and real-time movement analysis, we used the LABORAS system (Metris), designed to detect and analyze vibrations delivered from a cage to a carbon-fiber vibration-sensitive plate placed underneath the cage with a mouse. Each mouse was placed in the LABORAS cage without habituation. We recorded for 72 hours. The data from the last 48 hrs were analyzed by the software.

## Novel object recognition test

Object recognition test was performed in the open field box. On the first day, mice were allowed to explore two identical objects for 10 min. 24 hours later, mice were placed in the same box where one of the two objects was replaced with a new one. Exploration time for each object was measured. Object exploration was defined by the mouse’s nose being oriented toward the object and came within 2 cm of it as measured by EthoVision XT 10 program (Noldus).

## Rotarod assay

Mice were placed on the rotating rod for 10 sec, followed by the start of rod rotation. The rotating speed of rod was gradually increased from 4 to 40 rpm over 5 min. The assay was performed for 5 consecutive days, while measuring the latencies of mice falling from the rod or showing 360-degree rotation on the rod.

## Elevated plus maze

The elevated plus maze consisted of two open arms, two closed arms, and a center zone, and was elevated to a height of 50 cm above the floor. Mice were placed in the center zone and allowed to explore the space for 8 min. The data was analyzed using EthoVision XT 10 program (Noldus).

## Light-dark chamber test

The apparatus for light-dark test consisted of light (~200 lux) and dark (~0 lux) chambers adhered to each other. The size of the light chamber was 20 x 30 x 20 cm, and that of the dark chamber was 20 x 13 x 20 cm. An entrance enabled mice to freely move across the light and dark chambers. Mice were introduced to the center of the light chamber and allowed to explore the apparatus freely for 5 min. The time spent in dark and light chambers and the number of transitions were measured using EthoVision XT 10 program (Noldus).

## Three-chamber social interaction test

The size of the three-chambered apparatus ^6^ was 40 cm W x 20 cm H x 26 cm D with a center chamber of 12 cm W and side chambers of 14 cm W. In the first session, the mouse could freely move around the whole three-chambered apparatus with two small containers in the left or right corner for 10 min. The mouse was then gently confined in the center chamber while a novel ‘Object’ and a wild-type stranger mouse ‘Stranger 1 (129Sv strain)’ was placed in the two plastic containers. The subject mouse was then allowed to freely explore all three chambers for 10 min. In the third session, the subject mouse was again gently guided to the center chamber while the ‘Object’ was replaced with a wild-type ‘Stranger 2’ mouse. The subject mouse again freely explored all three chambers for 10 min.

## Morris water maze

Mice were trained to find the hidden platform (10 cm diameter) in a white plastic tank (120 cm diameter). Mice were given 3 trials per day with an inter-trial interval of 30 min. The learning phase the water maze of was performed for seven consecutive days, followed by the probe test on day 8 where mice were given 1 min to find the removed platform. For reversal training (days 9-13), the location of the platform was switched to the opposite position from the previously trained one, and mice were trained to learn the new position of the platform. Target quadrant occupancy and the exact number of crossings over the former platform location during the probe test were measured using EthoVision 10 program (Noldus).

## Fear conditioning test

All experiments were carried out in a fear conditioning system (Coulbourn Instruments). Training and testing were performed in a Plexiglas chamber with a stainless steel grid floor with constant illumination (50 lux). On the training day, mice were placed in the fear chamber and allowed to freely move the chamber for 2 min before they were received three foot shocks (2 sec, 0.8 mA, 1 min apart). To measure fear conditioning, mice were re-exposed the same chamber for 5 min without foot shock for 24 hours and 7 days after training.

**Seizure susceptibility**

WT and Ngl3^–/–(Hyb)^ mice received an intraperitoneal injection of pentylenetetrazol (PTZ) (40 mg/kg), or the same volume of saline, immediately before behavioral tests. For 10 min, mice were located at the novel cage with bedding (60 lux). To measure seizure susceptibility, behaviors of mice were analyzed by a blinded observer. Movement slowing (Phase 1), myoclonic jerk (Phase 2), and clonic and generalized tonic seizure (Phase 3), and death (Phase 4) were used as parameters. Seizure susceptibility score was according to a modified Racine scale ^7, 8^.

**Immunohistochemistry**

Mice were prepared at 3 weeks. After cardiac perfusion using 1% heparin and followed 4% PFA, brains were stored at PFA at least 1 day. Coronal sections (50 μm) were prepared using a vibratome (Leica). Sections were permeabilized with 0.5% TritonX-100 for 1 hour and incubated with primary antibodies (1:500, for both of vGluT1 and VGAT) for 24 hours. After washing with PBS 4 times, sections were incubated with appropriate secondary antibodies (Jackson Immunoresearch). After washing with PBS 2 times, slices were incubated with 1:1000 diluted Nissl (ThermoFisher) for 30 minutes to counterstain them. Every staining were proceed in 0.5% BSA, 0.3% Triton-X 100 in PBS. After washing with PBS 2 times, sections were mounted with Vectashield (Vector Laboratory) and images acquired on an LSM-780 (Zeiss). Images were analyzed using Metamorph 7.1(Molecular Devices), and cell layers of the hippocampus were distinguished manually. To measure the size of the region, 10-week-old brains were prepared as mentioned above. The shapes of each brain structure were manually marked, and the area was measured by a blinded observer. Only slices from -1.7~-1.82 from bregma were selectively analyzed.

**Fluorescent in situ hybridization**

Frozen sections (14 µm thick) were cut coronally through the hippocampal formation. Sections were thaw-mounted onto Superfrost Plus Microscope Slides (Fisher Scientific 12-550-15). The sections were fixed in 4% paraformaldehyde for 10 min, dehydrated in increasing concentrations of ethanol for 5 min, and finally air-dried. Tissues were then pretreated for protease digestion for 10 min at room temperature. For RNA detection, incubations with different amplifier solutions were performed in a HybEZ hybridization oven (ACDBio, CA, US) at 40 ^o^C. The probes used in this study were three synthetic oligonucleotides complementary to the nucleotide (nt) sequence 328 – 1408 of Mm-Ngl3/Lrrc4b-C1, nt 464-1415 of Mm- Slc17a7/Vglut1-C2, nt 1986-2998 of Mm-Slc17a6/Vglut2-C3, nt 62–3113 of Mm-Gad1-C3, nt 552–1506 of Mm-Gad2-C2 (ACDBio, CA, US). The labeled probes were conjugated to Atto 550 (C1), Alexa Fluor 488 (C2), and Atto 647 (C3). The sections were hybridized at 40 ^o^C with labeled probe mixtures (C1 + C2 + C3) per slide for 2 hours. Then the nonspecifically hybridized probes were removed by washing the sections, three times each in 1x wash buffer at room temperature for 2 min. Amplification steps involved sequential incubations at 40°C with Amplifier 1-FL for 30 min, Amplifier 2-FL for 15 min, Amplifier 3-FL for 30 min, and Amplifier 4 Alt B-FL for 15 min. Each amplifier solutions were removed by washing three times with 1x wash buffer for 2 min at RT. Fluorescent images were acquired using TCS SP8 Dichroic/CS (Leica), and the ImageJ program (NIH) was used to analyze the images.

**Supplementary references**

1. Han K*, et al.* Regulated RalBP1 binding to RalA and PSD-95 controls AMPA receptor endocytosis and LTD. *PLoS biology* **7**, e1000187 (2009).

2. Lie E*, et al.* SALM4 suppresses excitatory synapse development by cis-inhibiting trans-synaptic SALM3-LAR adhesion. *Nat Commun* **7**, 12328 (2016).

3. Song YS, Lee HJ, Prosselkov P, Itohara S, Kim E. Trans-induced cis interaction in the tripartite NGL-1, netrin-G1 and LAR adhesion complex promotes development of excitatory synapses. *Journal of cell science* **126**, 4926-4938 (2013).

4. Lee H, Lee EJ, Song YS, Kim E. Long-term depression-inducing stimuli promote cleavage of the synaptic adhesion molecule NGL-3 through NMDA receptors, matrix metalloproteinases and presenilin/gamma-secretase. *Philosophical transactions of the Royal Society of London Series B, Biological sciences* **369**, 20130158 (2014).

5. Gabriel LR, Wu S, Melikian HE. Brain slice biotinylation: an ex vivo approach to measure region-specific plasma membrane protein trafficking in adult neurons. *Journal of visualized experiments : JoVE*, (2014).

6. Silverman JL, Yang M, Lord C, Crawley JN. Behavioural phenotyping assays for mouse models of autism. *Nature reviews Neuroscience* **11**, 490-502 (2010).

7. Ferraro TN*, et al.* Mapping loci for pentylenetetrazol-induced seizure susceptibility in mice. *The Journal of neuroscience : the official journal of the Society for Neuroscience* **19**, 6733-6739 (1999).

8. Naydenov AV*, et al.* ABHD6 blockade exerts antiepileptic activity in PTZ-induced seizures and in spontaneous seizures in R6/2 mice. *Neuron* **83**, 361-371 (2014).
