# Supplementary figures and images for: NGL-3 in the regulation of brain development, Akt/GSK3b signaling, long-term depression, and locomotive and cognitive behaviors
Source: PLoS Biol. 2019 Jun 5;17(6):e2005326. doi: 10.1371/journal.pbio.2005326 (PMC6550391; doi:10.1371/journal.pbio.2005326)

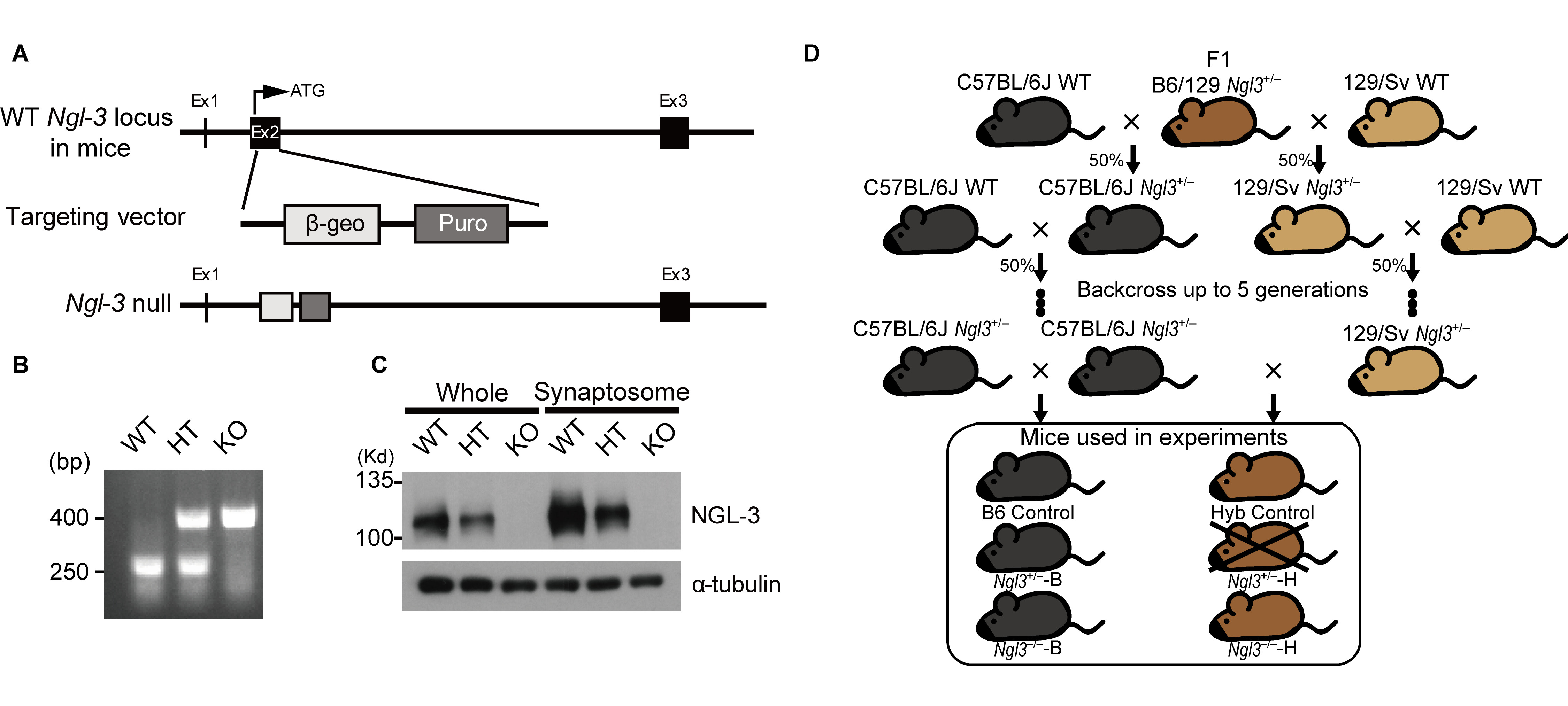

Supplement: S1 Fig — (A) Schematic diagram of the Ngl3 gene KO strategy. (B) PCR genotyping of Ngl3−/− mice. (C) Lack of detectable NGL-3 protein in whole-brain lysates of Ngl3−/−(B6) mice (8 weeks). (D) A schematic showing production of Ngl3−/− mice in two different genetic backgrounds: a pure C57BL/6J background (Ngl3−/−(B6)) and a hybrid 129/Sv + C57B/6J background (Ngl3−/−(Hyb)). HT, heterozygous; KO, knockout; NGL-3, Netrin-G ligand-3. (TIF) [file pbio.2005326.s004.tif]

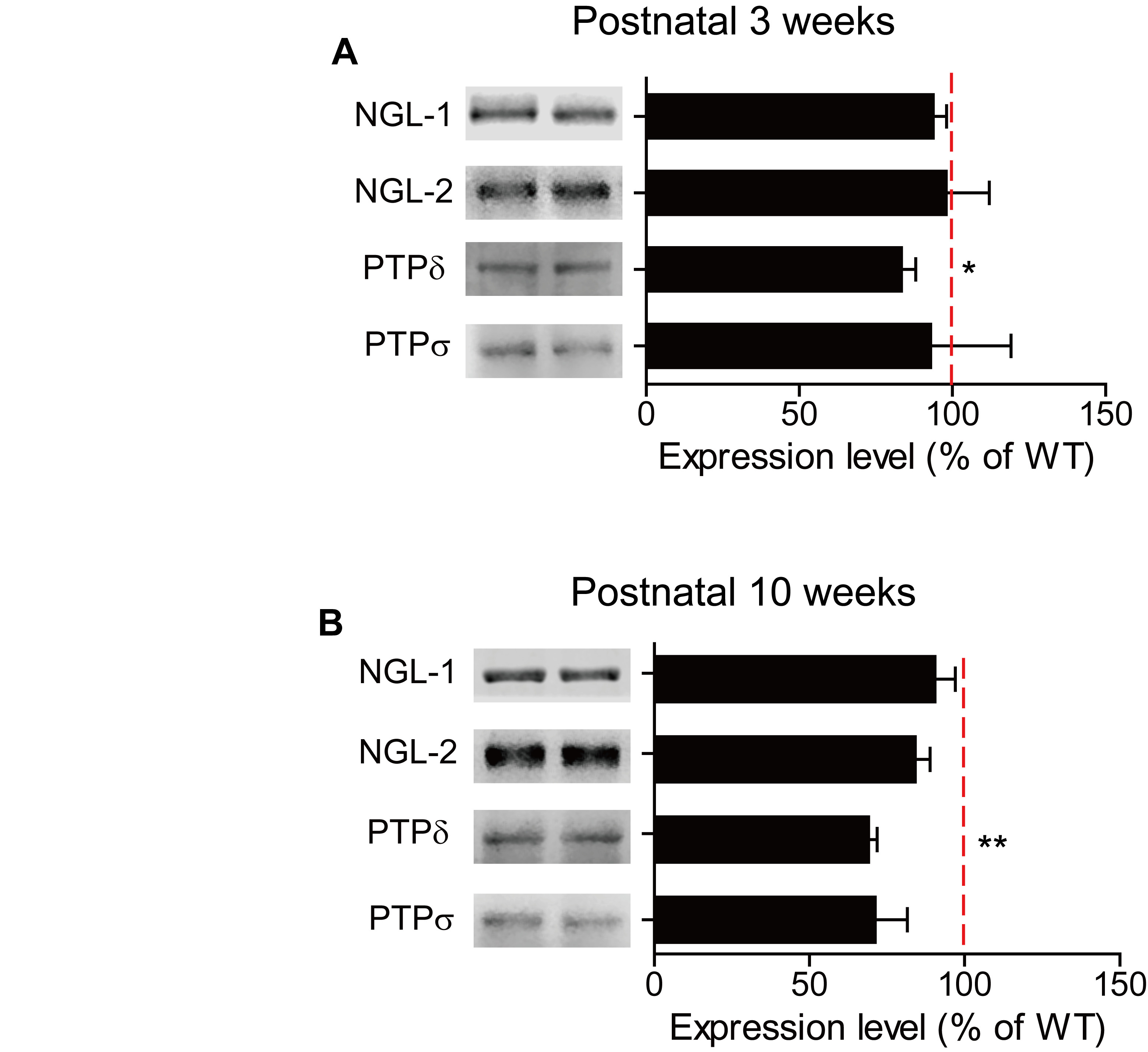

Supplement: S2 Fig — (A and B) Synaptic levels of NGL-3 relatives (NGL-1 and NGL-2) and NGL-3–binding presynaptic adhesion molecules (PTPδ and PTPσ) were also tested by immunoblot analysis of crude synaptosomes of the Ngl3−/− brain (3 and 10 weeks). Note that levels of PTPδ are significantly reduced at postnatal weeks 3 and 10. n = 4 mice for WT and KO, *P < 0.05, **P < 0.01, Student t test. Primary data can be found in S3 Data. KO, knockout; NGL, Netrin-G ligand; PTPδ, protein tyrosine phosphatase δ; PTPσ, protein tyrosine phosphatase σ; WT, wild-type. (TIF) [file pbio.2005326.s005.tif]

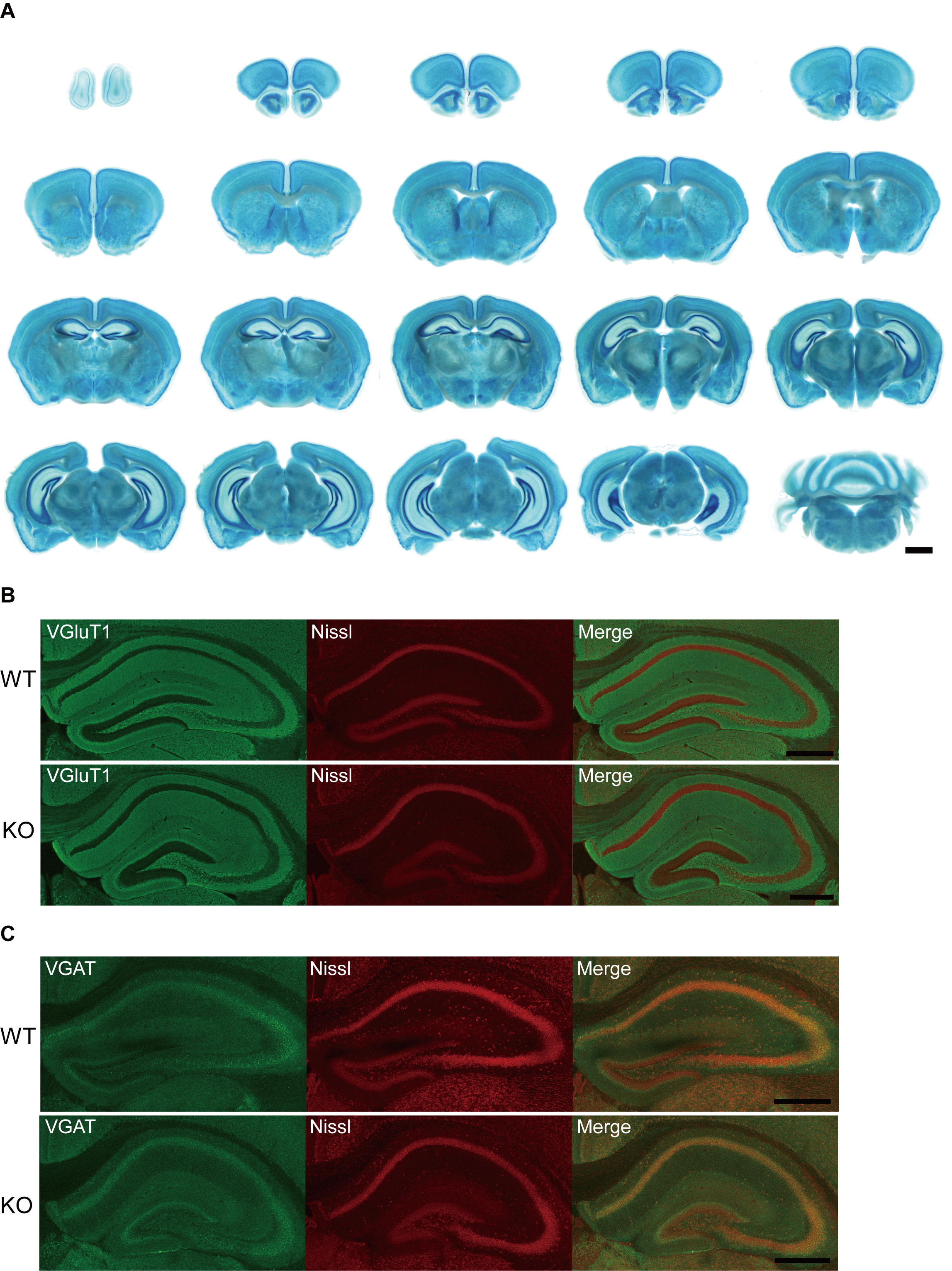

Supplement: S3 Fig — (A) Distribution patterns of NGL-3 proteins, revealed by X-gal staining of Ngl3+/–(Hyb) coronal brain slices (8–10 weeks). Scale bar, 1 mm. (B and C) Staining of VGluT1 (excitatory presynaptic marker) and VGAT (inhibitory presynaptic marker) in the hippocampus in Ngl3−/−(Hyb) mice (10 weeks). NGL-3, Netrin-G ligand-3; VGAT, vesicular GABA transporter; VGluT1, vesicular glutamate transporter 1. (TIF) [file pbio.2005326.s006.tif]

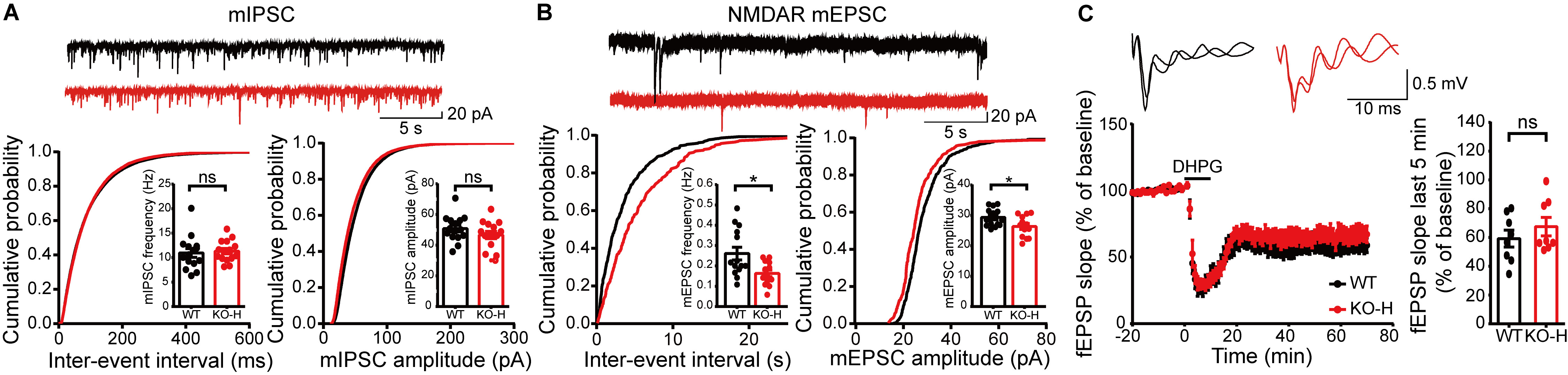

Supplement: S4 Fig — (A) Normal mIPSCs in hippocampal CA1 neurons of Ngl3−/−(Hyb) mice (P21–23). n = 15 cells from three mice for WT and KO; ns, not significant, Student t test. (B) Suppressed frequency and amplitude of NMDAR mEPSCs in hippocampal CA1 neurons of Ngl3−/−(Hyb) mice (P19–20). n = 14 cells from three mice for WT and 13, 3 for KO; *P < 0.05, Student t test. (C) Normal mGluR-LTD induced by DHPG treatment (50 μM) at hippocampal SC-CA1 synapses of Ngl3−/−(Hyb) mice (P16–20). n = 8, 4 for WT and 8, 3 for KO; ns, not significant, Student t test. Primary data can be found in S3 Data. CA1, Cornu Ammonis 1; DHPG, (RS)-3,5-dihydroxyphenylglycine; KO, knockout; mEPSC, miniature excitatory postsynaptic current; mGluR, metabotropic glutamate receptor; LTD, long-term depression; mIPSC, miniature inhibitory postsynaptic current; NMDAR, NMDA receptor; ns, not significant; P, postnatal day; SC-CA1, Schaffer collateral-CA1 pyramidal; WT, wild-type. (TIF) [file pbio.2005326.s007.tif]

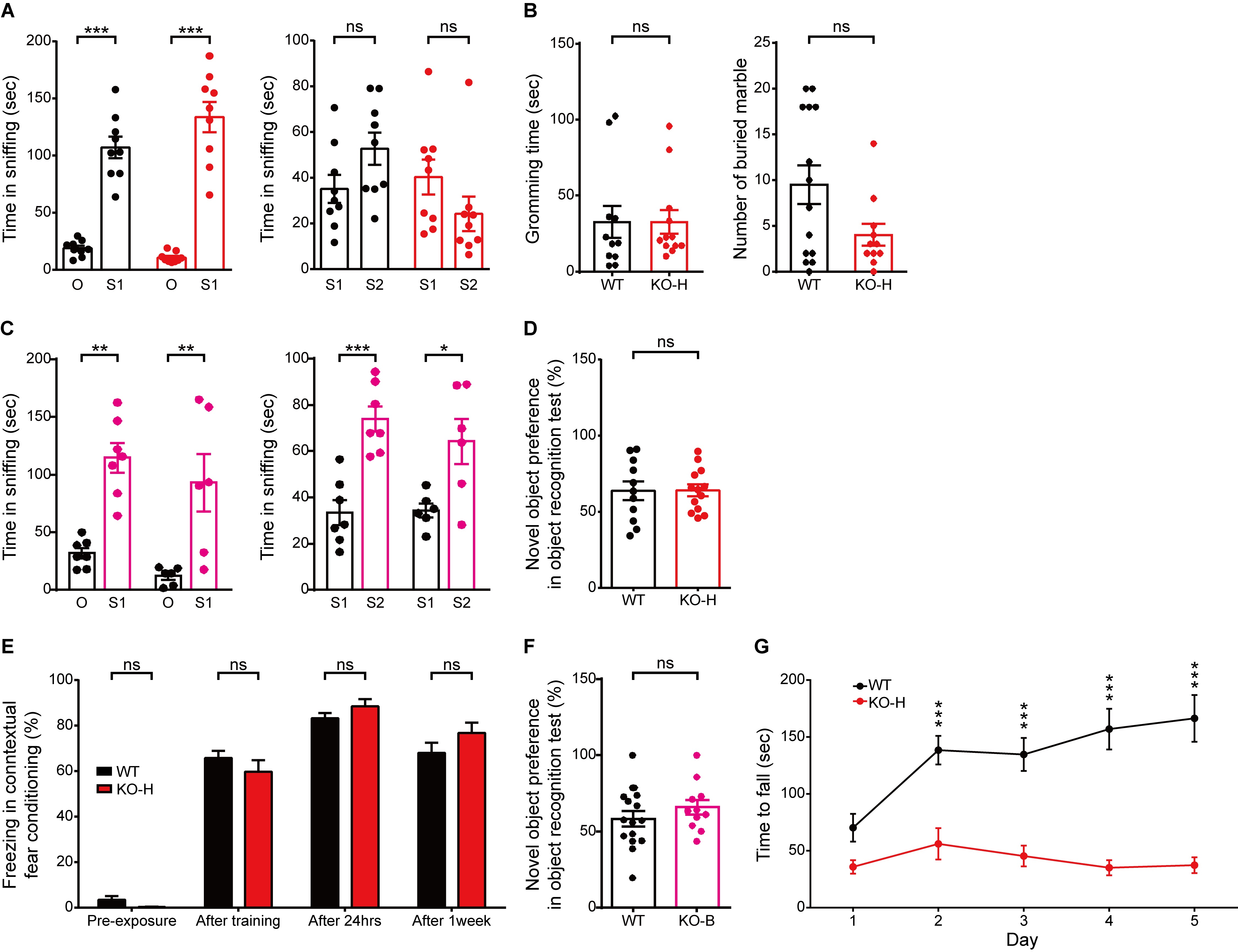

Supplement: S5 Fig — (A) Normal social interaction and social novelty recognition in Ngl3−/−(Hyb) mice (2–4 months) in the three-chamber social interaction test, as shown by time spent sniffing. n = 7 mice for WT and 6 for KO. ***P < 0.001, ns, not significant, one-way ANOVA with Tukey multiple comparison test. (B) Normal self-grooming and marble burying in Ngl3−/−(Hyb) mice (2–4 months). n = 11 mice for WT and 12 for KO for self-grooming, n = 14 mice for WT and 11 for KO for marble burying test; ns, not significant, Student t test. (C) Normal social interaction and social novelty recognition of Ngl3−/−(B6) mice (2–4 months) in the three-chamber social interaction test, as shown by time spent sniffing. n = 7 mice for WT and 6 for KO; *P < 0.05, **P < 0.01, ***P < 0.001, one-way ANOVA with Tukey test. (D) Normal object recognition memory in Ngl3−/−(Hyb) mice (2–4 months) in the novel-object recognition test. n = 15 mice for WT and 11 for KO; ns, not significant, Student t test. (E) Normal fear memory of Ngl3−/−(Hyb) mice (2–4 months) in the contextual fear conditioning test. n = 13 mice for WT and 10 for KO; ns, not significant, Student t test. (F) Normal object recognition of Ngl3−/−(B6) mice (2–4 months) in the novel-object recognition test. n = 15 mice for WT and 11 for KO; ns, not significant, Student t test. (G) Impaired motor learning of Ngl3−/−(Hyb) mice (2–4 months) in the rotarod test. n = 11 mice for WT and 12 for KO; ***P < 0.001, two-way ANOVA with Bonferroni test. Primary data can be found in S3 Data. KO, knockout; ns, not significant; WT, wild-type. (TIF) [file pbio.2005326.s008.tif]

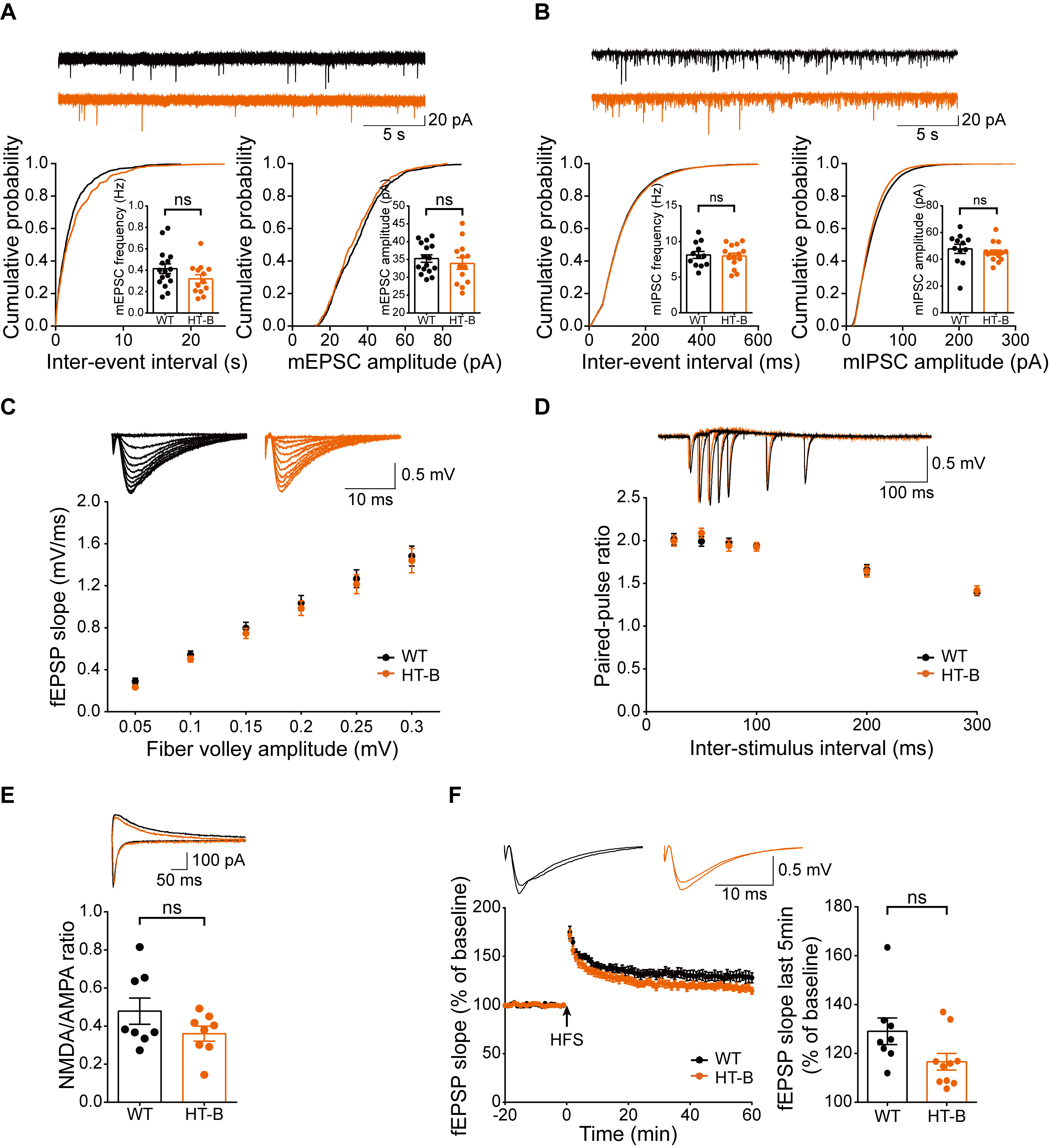

Supplement: S6 Fig — (A and B) Normal mEPSCs and mIPSCs in Ngl3+/–(B6) hippocampal CA1 neurons (P17–23 for mEPSCs and P18–22 for mIPSCs). n = 16 cells from five mice for WT and 13, 5 for KO (mEPSC); 12, 4 for WT and 15, 5 for KO (mIPSC); ns, not significant, Student t test. (C and D) Normal input-output relationship and paired-pulse ratio at Ngl3+/–(B6) hippocampal SC-CA1 synapses (P27–29), as shown by fEPSP slopes plotted against fiber volley amplitudes and paired-pulse ratios plotted against inter-pulse intervals. n = 10 cells from three mice for WT and KO for both input-output and paired-pulse ratio, two-way ANOVA with Bonferroni test. (E) Normal NMDAR function at Ngl3+/–(B6) hippocampal SC-CA1 synapses (P17–23), as shown by the NMDA/AMPA ratio. n = 8 cells from five mice for WT and 8, 7 for KO; ns, not significant, Student t test. (F) Normal LTP induced by HFS (100 Hz, 1 second) at Ngl3+/–(B6) hippocampal SC-CA1 synapses (P23–33). n = 8 slices from six mice for WT and 10, 7 for KO; ns, not significant, Student t test. Primary data can be found in S3 Data. AMPA, α-amino-3-hydroxy-5-methyl-4-isoxazolepropionic acid; CA1, Cornu Ammonis 1; fEPSP, field excitatory postsynaptic potential; KO, knockout; LTP, long-term potentiation; mEPSC, miniature excitatory postsynaptic current; mIPSC, miniature inhibitory postsynaptic current; NMDA, N-methyl-D-aspartate; NMDAR, NMDA receptor; P, postnatal day; SC-CA1, Schaffer collateral-CA1 pyramidal; WT, wild-type. (TIF) [file pbio.2005326.s009.tif]

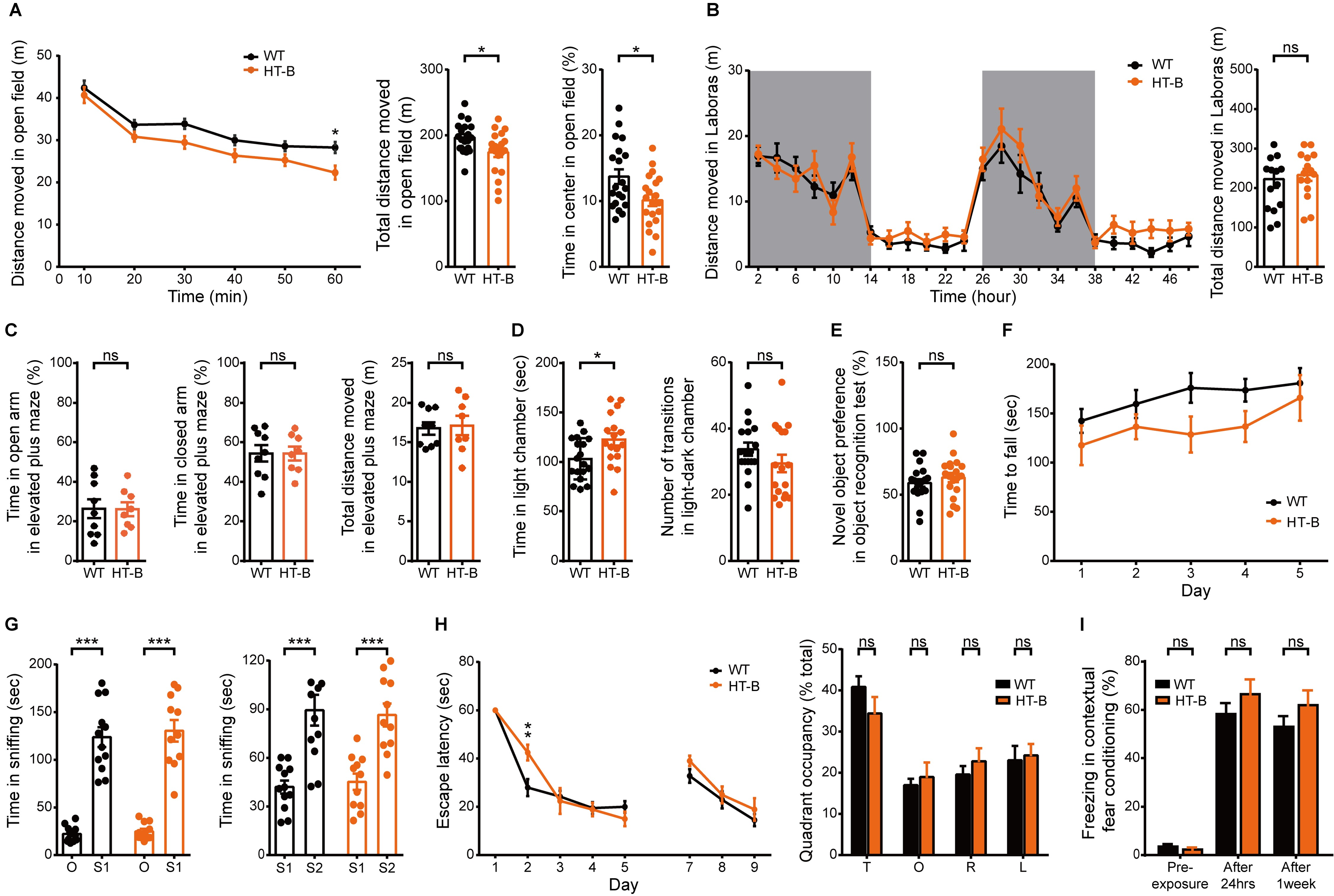

Supplement: S7 Fig — (A) Moderate hypoactivity of Ngl3+/–(B6) mice (2–4 months) in the open-field test. Mean ± SEM. n = 20 mice for WT and heterozygote (HT); *P < 0.05, two-way ANOVA with Bonferroni test and Student t test. (B) Normal locomotor activity of Ngl3+/–(B6) mice (2–4 months) in the Laboras test, in which mouse movements are continuously monitored for 48 hours. n = 15 mice for WT and HT; ns, not significant, two-way ANOVA with Bonferroni test and Student t test. (C) Normal anxiety-like behavior of Ngl3+/–(B6) mice (2–4 months) in the elevated plus maze test, as shown by time spent in and entries into open/closed arms. n = 9 mice for WT and 8 for HT; ns, not significant, Student t test. (D) Moderate anxiolytic-like behavior of Ngl3+/–(B6) mice (2–4 months) in the light-dark test, as shown by transition number and chamber time. n = 18 mice for WT and 17 for HT; *P < 0.05, ns, not significant, Student t test. (E) Normal object memory of Ngl3+/–(B6) mice (2–4 months) in the novel-object recognition test. n = 20 mice for WT and 19 for HT; ns, not significant, Student t test. (F) Normal motor learning of Ngl3+/–(B6) mice (2–4 months) in the rotarod test. n = 10 mice for WT and HT; ns, not significant, repeated measure of ANOVA. (G) Normal social interaction and social novelty recognition of Ngl3+/–(B6) mice (2–4 months) in the three-chamber social interaction test, as shown by time spent in sniffing. n = 12 mice for WT and 10 for HT; ***P < 0.001, one-way ANOVA with Tukey test. (H) Normal spatial memory of Ngl3+/–(B6) mice (2–4 months) in the learning, probe, and reversal phases of the Morris water maze test. Quadrant occupancy during the probe phase is also indicated. n = 10 mice for WT and 9 for HT; **P < 0.01, ns, not significant, two-way ANOVA with Bonferroni test and Student t test. (I) Normal fear memory of Ngl3+/–(B6) mice (2–4 months) in the contextual fear conditioning test. n = 12 mice for WT and KO; ns, not significant, Student t test. Primary data can be found in S3 Data [file pbio.2005326.s010.tif]

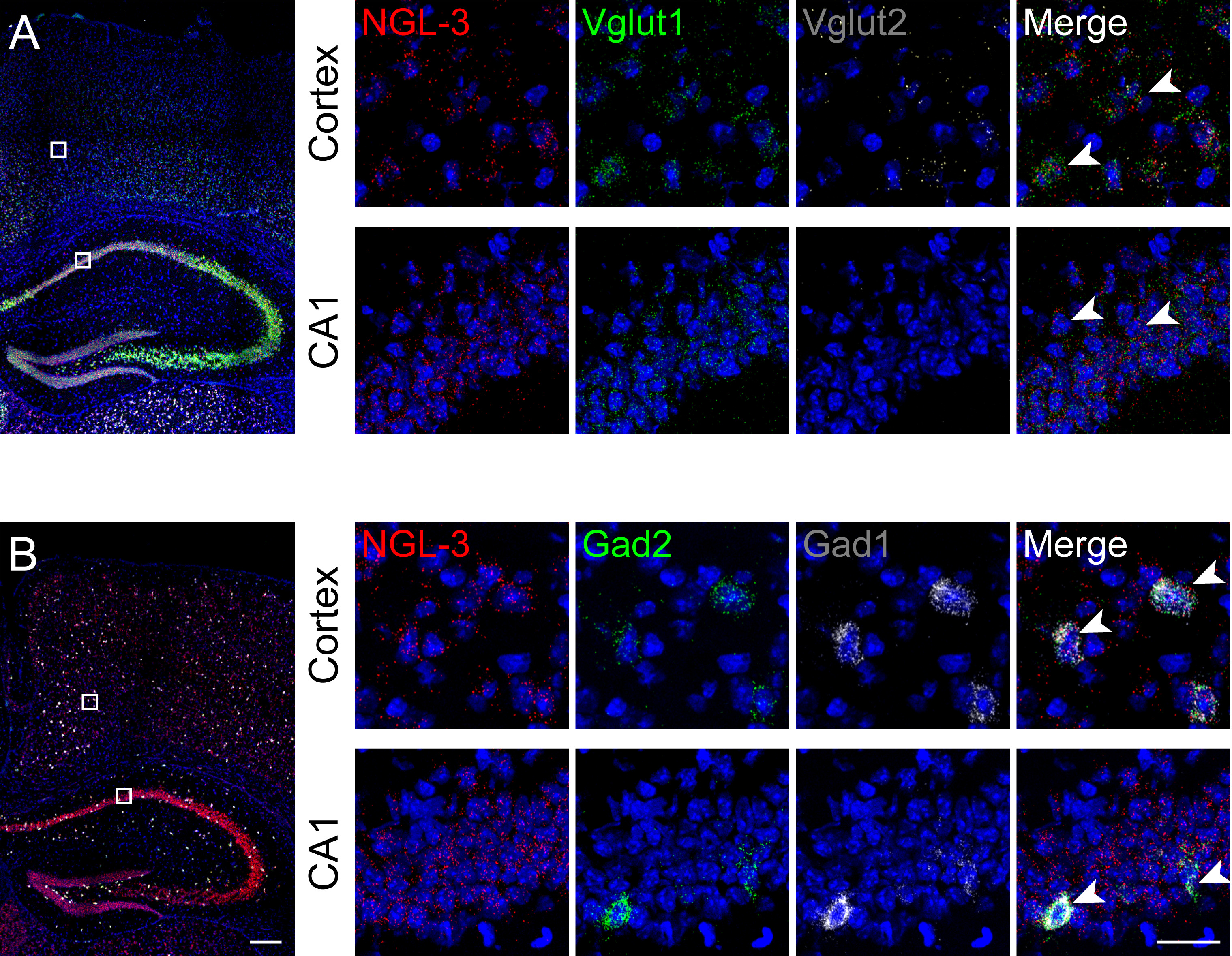

Supplement: S8 Fig — (A and B) Localization of Ngl3/Lrrc4b mRNAs in both excitatory and inhibitory neurons in the cortex and hippocampus of WT mice (P56), as determined by fluorescence in situ hybridization and shown by the colocalization of Ngl3/Lrrc4b mRNAs and Vglut1/2 (excitatory neuronal marker) or Gad1/2 (inhibitory neuronal marker). Arrowheads indicate examples of neurons that express both Ngl3/Lrrc4b and Vglut1/2 or Gad1/2 mRNAs. Scale bar, 0.2 mm (left) and 20 μm (right). Gad1/2, glutamate decarboxylase 1/2; Ngl3/Lrrc4b, Netrin-G ligand-3/Leucine-rich repeat-containing protein 4B; P, postnatal day; Vglut1/2, vesicular glutamate transporter 1/2; WT, wild-type. (TIF) [file pbio.2005326.s011.tif]

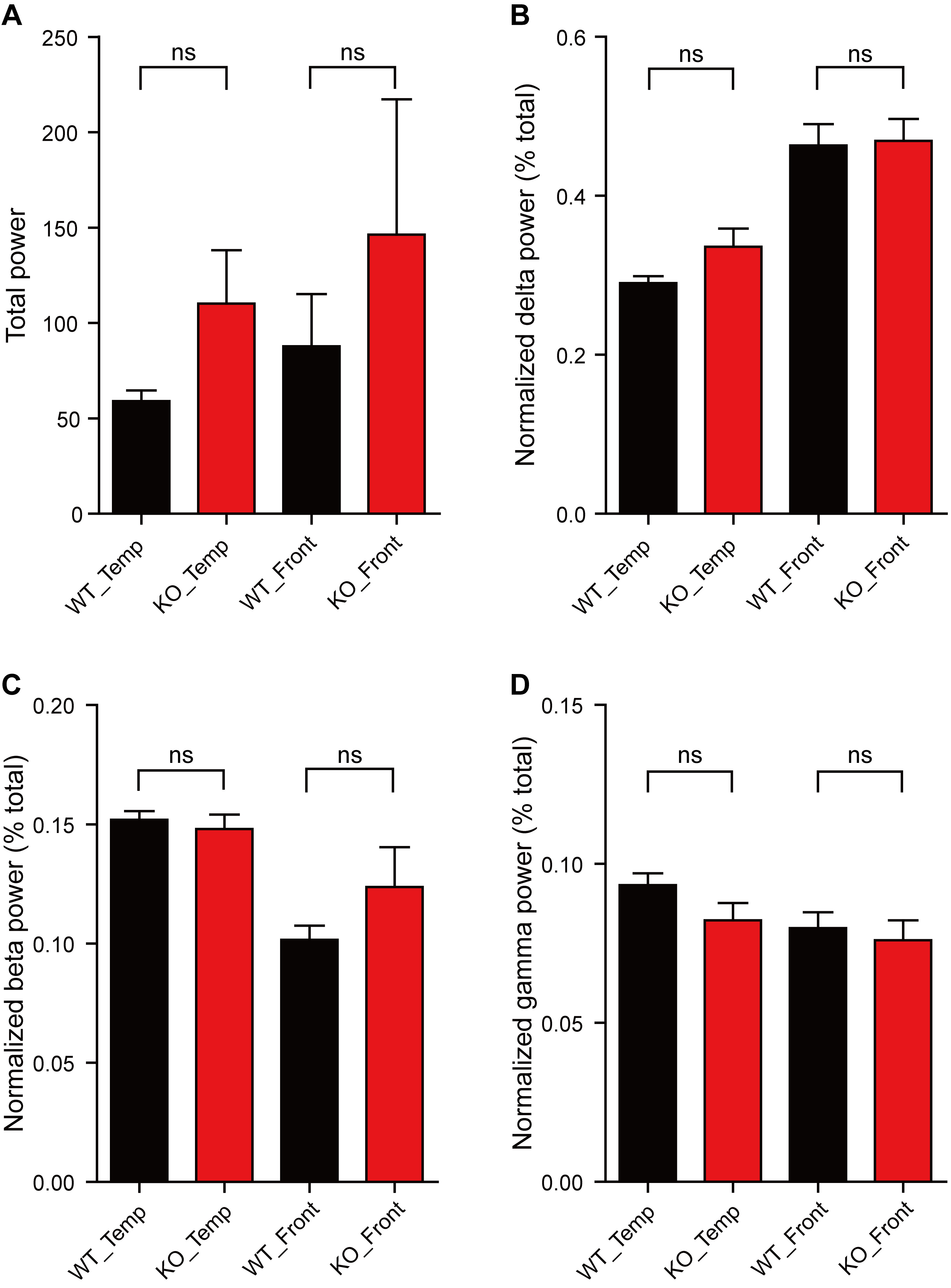

Supplement: S9 Fig — (A-D) Total power of brain oscillations (A) and brain oscillations in different frequency ranges, normalized to the total power (B–D). Note that none of the comparisons yielded significant differences except alpha and theta ranges in the temporal lobe (see main figure panels). n = 6 mice for WT and KO; ns, not significant, Student t test. Primary data can be found in S3 Data. KO, knockout; ns, not significant; WT, wild-type. (TIF) [file pbio.2005326.s012.tif]

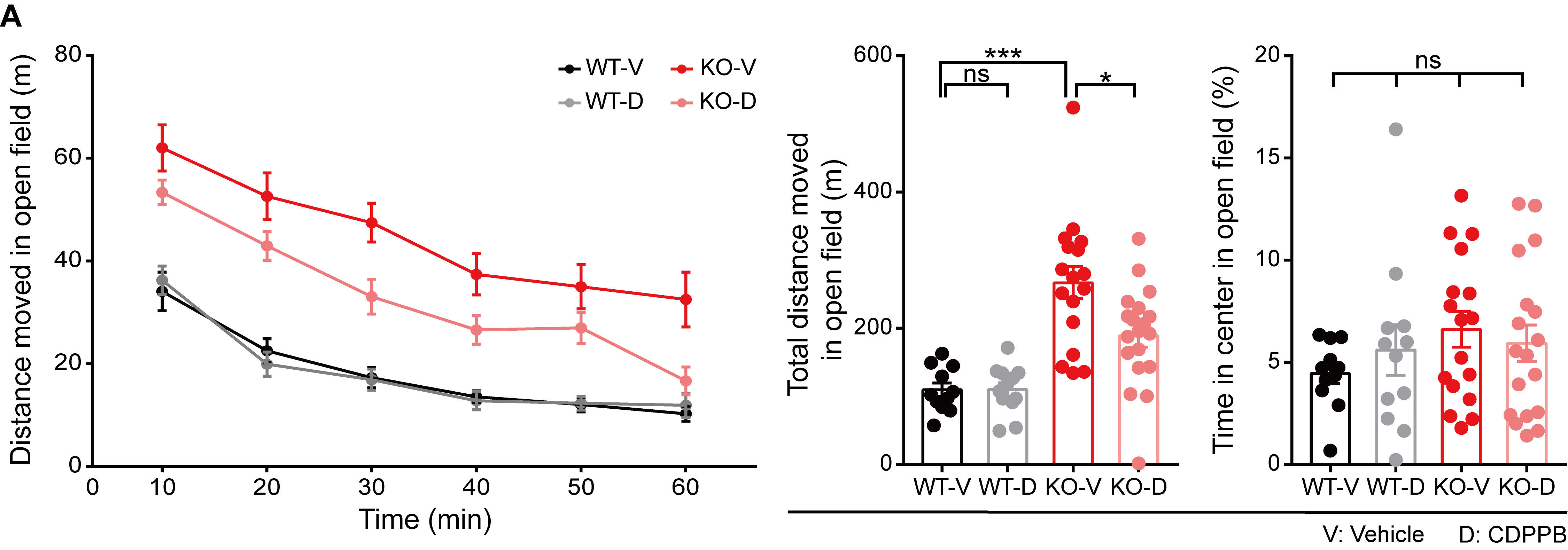

Supplement: S10 Fig — (A) CDPPB (10 mg/kg, intraperitoneal), administered 30 minutes before the test, rapidly rescues the hyperactivity of Ngl3−/− mice (2–3 months) in the open-field test, as shown by the distance moved. n = 11 mice for WT-saline (V), 12 for WT-DCS (D), 17 for KO-V, and 18 for KO-D; *P < 0.05, ***P < 0.001, ns, not significant, two-way ANOVA with Bonferroni test. Primary data can be found in S3 Data. DCS, D-cycloserine; KO-D, knockout, drug; KO-V, knockout, vehicle; ns, not significant; V, vehicle; WT, wild-type. (TIF) [file pbio.2005326.s013.tif]
